# Characterization of Acetonitrile Isotopologues as Vibrational Probes of Electrolytes

**DOI:** 10.1021/acs.jpcb.1c09572

**Published:** 2021-12-28

**Authors:** Bogdan Dereka, Nicholas H. C. Lewis, Jonathan H. Keim, Scott A. Snyder, Andrei Tokmakoff

**Affiliations:** † James Franck Institute, The University of Chicago, Chicago, Illinois 60637, United States; ‡ Department of Chemistry, The University of Chicago, Chicago, Illinois 60637, United States; § Institute for Biophysical Dynamics, The University of Chicago, Chicago, Illinois 60637, United States; ∥ Joint Center for Energy Storage Research, Argonne National Laboratory, Lemont, Illinois 60637, United States

## Abstract

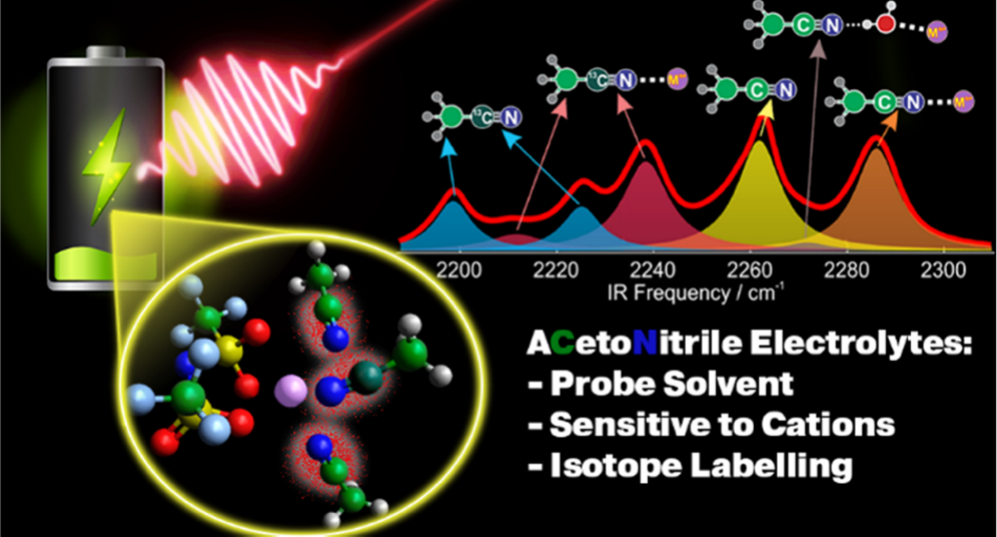

Acetonitrile has emerged as a solvent candidate for novel electrolyte formulations in metal-ion batteries and supercapacitors. It features a bright local C≡N stretch vibrational mode whose infrared (IR) signature is sensitive to battery-relevant cations (Li^+^, Mg^2+^, Zn^2+^, Ca^2+^) both in pure form and in the presence of water admixture across a full possible range of concentrations from the dilute to the superconcentrated regime. Stationary and time-resolved IR spectroscopy thus emerges as a natural tool to study site-specific intermolecular interactions from the solvent perspective without introducing an extrinsic probe that perturbs solution morphology and may not represent the intrinsic dynamics in these electrolytes. The metal-coordinated acetonitrile, water-separated metal–acetonitrile pair, and free solvent each have a distinct vibrational signature that allows their unambiguous differentiation. The IR band frequency of the metal-coordinated acetonitrile depends on the ion charge density. To study the ion transport dynamics, it is necessary to differentiate energy-transfer processes from structural interconversions in these electrolytes. Isotope labeling the solvent is a necessary prerequisite to separate these processes. We discuss the design principles and choice of the CD_3_^13^CN label and characterize its vibrational spectroscopy in these electrolytes. The Fermi resonance between ^13^C≡N and C–D stretches complicates the spectral response but does not prevent its effective utilization. Time-resolved two-dimensional (2D) IR spectroscopy can be performed on a mixture of acetonitrile isotopologues and much can be learned about the structural dynamics of various species in these formulations.

## 1 Introduction

Sustainable energy is one of the most urgent needs in modern society. Renewable sources have been steadily increasing their share in the energy production market in recent years.^1^ However, the intermittent nature of solar and wind energy harvesting requires the means of grid energy storage to cope with the imbalances in energy production and consumption.^2^ The rise of portable electronics demands powerful, light, and affordable sources to power these devices. Battery energy storage technologies, especially Li-ion batteries (LIB), have been at the forefront of the efforts to meet these challenges. The current state of LIB has almost reached its limit in energy density, and future innovations are needed to ramp up the capacity, safety, rate performance, electrochemical stability, lifespan, and reduce the cost of future batteries.^3^ Combined with the limited and costly supply of the raw materials for LIBs,^4,5^ as well as ethical concerns related to their mining,^6^ trailblazing metal-ion battery technologies emerge based on abundant and cheap metal ions serving as charge carriers, such as Zn^2+^,^7^ Mg^2+^,^8^ and Ca^2+^.^9^ The path beyond Li ions dwells on the development of new chemistries for electrolyte formulations: new salts,^10^ additives,^3,10^ revisited or exotic solvents,^3,11^ and exploration of unusual concentration regimes, such as superconcentration.^12−14^ Understanding the microscopic structure and dynamics occurring on a wide range of time scales in these novel electrolytes is a prerequisite for revealing the functional correlations between the formulation content and its performance and will aid in the data-driven design of future electrolytes.

Out of many methods that are fruitfully utilized to reveal various aspects of the microscopic behavior of electrolytes, we focus on infrared (IR) spectroscopy. It is an intrinsically structure-specific method that probes molecular vibrations, and time-resolved IR can achieve femtosecond time resolution, thus opening the way to probe the dynamical aspect of structural variations on the time scale of basic molecular motions. Molecules that contain local vibrational markers, that is, few-atom molecular arrangements yielding a spectrally isolated site-specific vibrational signal decoupled from the rest of the environment, present a useful strategy to probe local structural arrangements in liquids. These vibrational probes must satisfy several conditions to be useful for interrogation of liquid battery electrolytes: (i) their spectral characteristics should be sensitive to their environment, and specifically to interactions with ions; (ii) they should not perturb the intrinsic structure of the electrolyte; and (iii) for time-resolved studies, excited vibrations should be long lived enough to allow access to a wide temporal window. The first criterion is reasonably facile to accommodate, as multiple employed markers,^15^ such as C=O,^16−18^ C≡N,^19−25^ or N≡C^–^,^26,27^ N_3_^–^,^28−31^ O–H,^32−34^ and others,^27,35^ have been demonstrated to sense changes in their immediate environment. The second requirement of the nonperturbative nature of the vibrational reporter represents a challenge and is usually implicitly assumed to be fulfilled. It might be a sensible assumption in some cases,^36−38^ such as in dilute solutions when the introduction of extrinsic reporters dramatically extends the available temporal range,^39^ but extrinsic probes cannot generally be considered innocent. The problem is augmented in unconventional systems, such as superconcentrated (“solvent-in-salt”) electrolytes, where there are as many, or even fewer, solvent molecules as ions, no free bulk-like solvent remains, and the structure is highly coordinated and tightly packed. The introduction of the extrinsic probe molecule will necessarily intrude into the native ion-molecular arrangements but not necessarily sample the entire range of structures and interactions within the liquid.^40,41^ Potentially, even more complicated situation might be envisioned in highly concentrated electrolytes containing several salts (“solvent-in-bisalt”)^42,43^ or in so-called locally superconcentrated electrolytes—multicomponent systems comprising ions, solvating solvent, and nonsolvating diluent that are hypothesized to feature domains of clustered ions solvated by interacting solvent molecules and spread apart by the nonsolvating diluent.^44^ The third requirement imposed by time-resolved IR is one of the most challenging as typical lifetimes of vibrational chromophores are on the order of several picoseconds, thus limiting the accessible range of time scales up to 10–20 ps and making the selection of usable probes rather limited. It largely motivated the incorporation of extrinsic long-lived species that take advantage of vibrational decoupling by introducing insulating heavy atoms between the vibrational reporter and the rest of the molecule (e.g., thio-^41,45,46^ and selenocyanates^39,45−47^) that dramatically slows down vibrational relaxation.

In the current work, we avoid the extrinsic-probe approach by utilizing the solvent vibrational marker mode and demonstrate its applicability to probe ion–solvent interactions within various electrolytes. Specifically, we use the acetonitrile (ACN) C≡N stretch with a variety of salts composed of battery-relevant cations and the same noncoordinating anion across the entire possible range of concentrations from the dilute to the superconcentrated regime. Acetonitrile (ACN) has recently emerged as a promising solvent candidate for new generations of battery electrolytes due to its excellent oxidative stability and compatibility with 5 V-class cathodes, high dielectric constant, low viscosity, melting point, and price, as well as moderate toxicity. On top of these characteristics, one of the metrics of utmost importance in metal-ion batteries is solvent donicity (donor number)—an empirical measure of electron-donating ability and Lewis basicity of a coordinating nucleophilic solvent, which determines the strength of its interaction with metal ions.^48^ The major hurdle that precluded ACN utilization in batteries in the past is its poor reductive stability that can now be overcome in the superconcentrated regime due to the formation of the protective layer of the anion-derived interphase on the anode.^49,50^ Thus, acetonitrile is a typical representative of solvents that have been conventionally deemed exotic in the context of battery media but that have been rejuvenized due to the recent advancement of superconcentration.^3^ Except for IR spectroscopy of water,^32,34,51^ looking at a solution from the solvent perspective is not a common spectroscopic approach; despite being an intrinsic reporter naturally present in the system, which is highly desirable, it comes with its own shortcomings. Solvent concentration is always high even in the most concentrated salt solutions, which except for necessitating thin path lengths otherwise plays a little role in stationary IR experiments. However, it can be detrimental for time-resolved IR spectroscopy because it facilitates the intermolecular vibrational energy transfer (VET).^52^ VET can mask other dynamical events, such as a chemical exchange. Some of the recent time-resolved IR studies have interrogated the solvent modes of organic carbonates,^53−57^ ureas,^58^ and acetonitrile^59^ in Li^+^ electrolytes, but they could not discern the competing VET and chemical exchange pathways. One of the common ways to combat this issue is by introducing isotope-edited solvent molecules. It has been a standard approach for a long time to study the water dynamics by doping H_2_O/D_2_O with a small fraction of D/H, respectively.^51,60,61^ Here, we discuss the design of various acetonitrile isotopologues and analyze their applicability to probing liquid battery electrolytes with IR spectroscopy. We demonstrate that despite the complications arising from the Fermi resonance (FR), CD_3_^13^CN in mixture with CD_3_CN is the most viable option for studying site-specific ion–solvent interactions in acetonitrile electrolytes from the solvent perspective.

## 2 Experimental Section

### 2.1 Materials

KTFSI, LiTFSI, Mg(TFSI)_2_, Ca(TFSI)_2_, Zn(TFSI)_2_, Ba(TFSI)_2_ salts, and 18-crown-6 macrocyclic ether were purchased from Sigma-Aldrich and used as received. CD_3_CN (99.8% D) was purchased from Cambridge Isotope Laboratories in sealed 1 mL glass ampules. CH_3_CN (extra dry) was obtained from Sigma-Aldrich and stored under the sealed septum above the molecular sieves. ^13^CH_3_^13^CN (99% ^13^C) was purchased from Cambridge Isotope Laboratories in a sealed glass ampule.

CD_3_^13^CN was synthesized by the reaction of potassium cyanide(^13^C) with iodomethane-d_3_ (CD_3_I) using a modified procedure from ref (62). The synthesis and characterization procedures are presented in the Supporting Information (SI).

All solutions were prepared by weighing a specific amount of salt, adding solvent, and mechanically stirring and sonicating the mixture until a clear solution was formed. No heating was allowed to facilitate the dissolution process to avoid unwanted reactions with oxygen and moisture in the atmosphere as well as the possible transition into the thermodynamically unstable supercooled regime for superconcentrated electrolytes upon return to the room temperature.^63^ Solution compositions are presented in the SI (Table S1).

### 2.2 Experimental Methods

Stationary IR spectra were measured on a Bruker Tensor 27 Fourier transform infrared (FTIR) spectrometer in a transmission mode. The samples were held between two 1 mm thick CaF_2_ windows in a brass cell without a spacer. An average of 64 individual spectra with a spectral resolution of 1 cm^–1^ was obtained for each measurement.

A two-dimensional (2D) IR spectrometer based on a pulse shaper design is described in ref (18). To summarize, a 1 kHz Ti:Sapphire regenerative amplifier (Spectra-Physics, Solstice, 800 nm, 90 fs pulse duration) pumps an optical parametric amplifier (Light Conversion, TOPAS Prime) equipped with a difference frequency generation module (Light Conversion, NDFG) to produce mid-IR pulses centered at 2250 cm^–1^ with ∼300 cm^–1^ bandwidth at full width at half-maximum with 100 fs duration and >20 μJ per pulse. The probe beam was obtained by the front face reflection off an uncoated wedged CaF_2_ window. The pump was split into a pair of pulses with a 4-*f* mid-IR pulse shaper (PhaseTech QuickShape). The time delay τ_1_ and phase difference between these pulses were controlled via a home-written LabView software. For 2D IR measurements, we scanned τ_1_ from 0 to −5 ps in 33 fs steps using a rotating frame at 1900 cm^–1^ and a 2 × 2 phase cycling scheme. The excitation axis was resolved by numerically Fourier transforming the data along τ_1_ after windowing and zero padding. The pump was compressed by optimizing the second harmonic generation in the AgGaS_2_ nonlinear crystal as a function of the second-, third-, fourth-, fifth-, and sixth-order dispersion applied by the pulse shaper and was verified by the interferometric autocorrelation. Typically, the dispersion compensation above the third order was negligible. The cross-correlation of the pump and probe pulses in the sample was about 150 fs full width at half-maximum and was verified with a nonresonant response of a CaF_2_ window and a Si wafer. The sample was held between two 200 μm thin CaF_2_ windows with or without a mylar spacer (depending on the sample) tightly wrapped with Parafilm to prevent evaporation. Spacer thickness of 3.6 and 6 μm was employed. The change in probe light absorption induced by the pump was determined using a spectrograph (Horiba Triax 190, 300 lines/mm grating) and detected with a 64 pixel HgCdTe array (Infrared Associates MCT-7–64, Infrared Systems Development IR-6416), thus determining the detection axis ω_3_. One or two grating positions were acquired depending on the sample. Adjacent spectral regions had overlapping portions to facilitate stitching of the spectral data. Spectral resolution along the detection axis was 0.6–0.7 cm^–1^. The polarization dependence was determined by rotating the polarization of the pump to 45° relative to the probe using a λ/2 waveplate and rotating the analyzer in front of the spectrometer to obtain the parallel (*S*_∥_) and perpendicular (*S*_⊥_) contributions to the signal. The isotropic signal was subsequently determined as *S*_iso_ = (*S*_∥_ + 2 × *S*_⊥_)/3. All spectra in this work correspond to isotropic signals.

## 3 Results and Discussion

### 3.1 Stationary IR Spectroscopy of Acetonitrile Electrolytes

**Figure 1:**
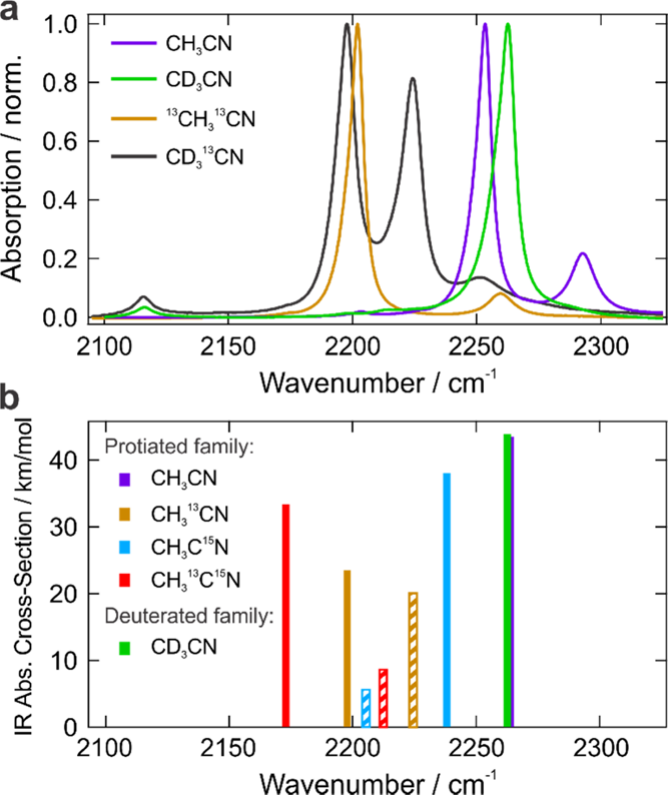
Stationary IR spectra of several acetonitrile isotopologues. (a) Experimental IR spectra of neat liquids of isotopologues from different families presented in Table 1. (b) Anharmonic frequencies and intensities calculated for the protiated family and CD_3_CN in the 2150–2350 cm^–1^ spectral region around the C≡N stretch absorption. Combination bands that borrow the intensity from the bright C≡N stretch band are depicted as white-striped bars with the same color as the band they are borrowing from.

The acetonitrile molecule features a prominent local C≡N stretching vibrational mode that presents an opportunity to use IR spectroscopy to study its interactions with electrolyte salts from the solvent perspective without introducing any extrinsic reporters. However, using the acetonitrile C≡N spectroscopy in electrolytes is complicated by the Fermi resonance (FR) between this mode and nearby dark transitions. For example, in conventional CH_3_CN, the C≡N band is downshifted to 2253 cm^–1^ and a combination band of the C–C stretching and C–H bending modes^64^ emerges at 2293 cm^–1^, whereas in CD_3_CN, where there is no FR, the C≡N transition is the only prominent band in this region peaking at 2262 cm^–1^ (Figure 1a). As a result, for spectroscopy, we use the readily accessible CD_3_CN as the main acetonitrile isotopologue.

**Figure 2:**
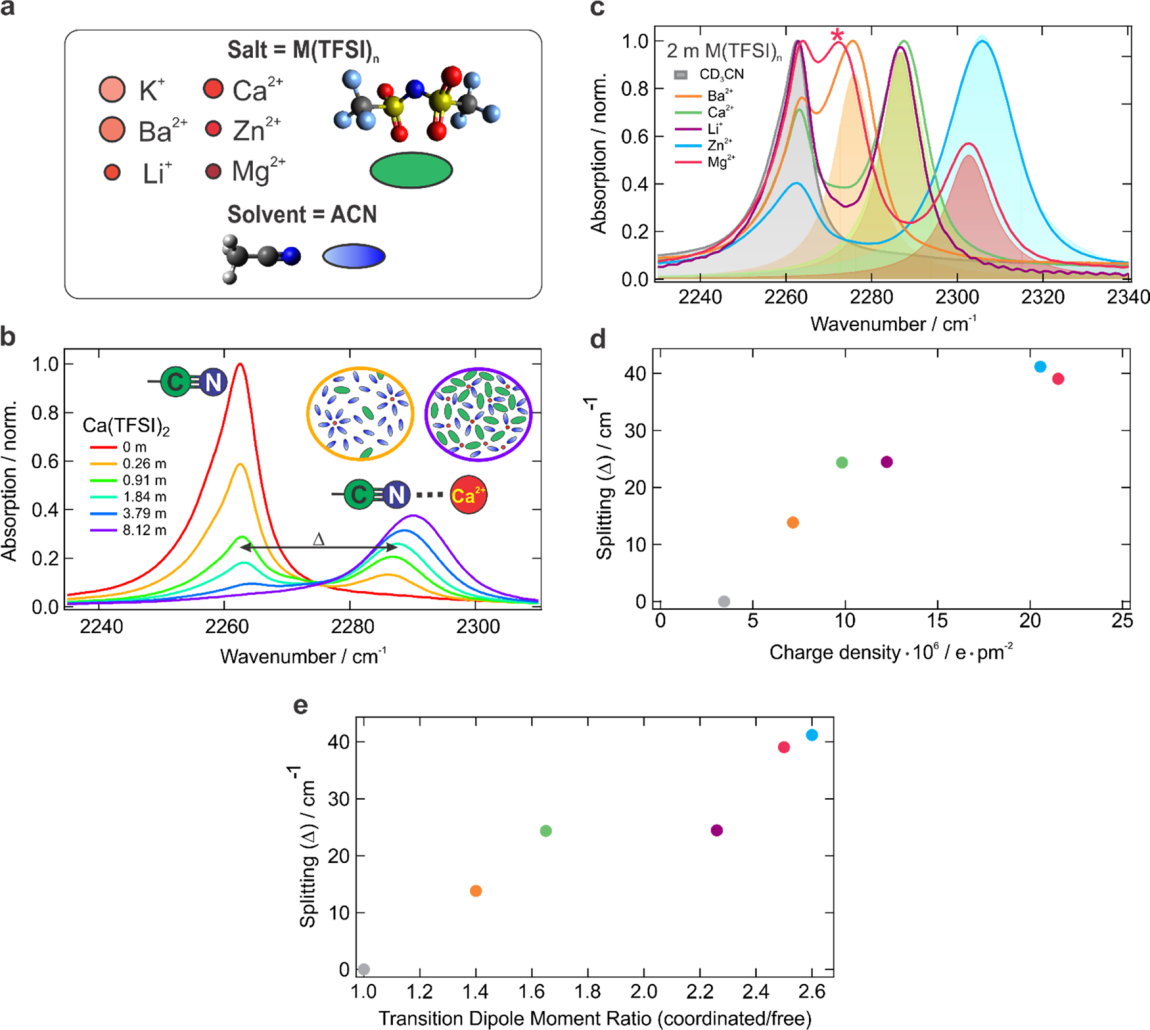
Stationary IR spectroscopy of the CD_3_CN solvent in acetonitrile electrolytes. (a) M(TFSI)*_n_* salts in acetonitrile serve as electrolytes. Various solution constituents are schematically depicted as shown. Color saturation of cations reflects the increasing charge density, whereas the circle size is proportional to the ionic radius. (b) Solvent C≡N stretch region in the presence of various quantities of Ca(TFSI)_2_ salt. Assignments of two IR bands are indicated. Spectral splitting (Δ = ν_ACN···M_^*n*+^ – ν_ACN_) between the cation coordinated and free C≡N band maxima is marked with an arrow. Schematic depictions of the solution structure in the dilute (orange) and superconcentrated (purple) regimes are shown. (c) IR spectra in the C≡N stretch region in 2 m M(TFSI)*_n_* salt solutions. The metal-coordinated C≡N band is shaded with color; an uncoordinated solvent is gray shaded. The asterisk denotes the band corresponding to the nitrile H-bonded to Mg^2+^-coordinated water. (d) Spectral splitting is determined by the cation charge density. (e) Splitting and transition dipole moment scaling are strongly correlated.

To introduce metal ions in the solution, we use M(TFSI)*_n_* salts, where M^*n*+^ represents a series of cations of increasing charge density (K^+^, Ba^2+^, Li^+^, Ca^2+^, Zn^2+^, Mg^2+^) and TFSI^–^ is a bis(trifluoromethylsulfonyl)imide anion (Figure 2a). TFSI salts have been attracting significant interest for the exploration of novel electrolyte formulations due to the noncoordinating nature of the bulky anion^65^ and their exceptional solubility in a variety of solvents opening the possibility to venture into the superconcentrated regime.^11,49,66−68^ Various aspects of the solution structure, morphology, and transport properties of LiTFSI/ACN solutions have been investigated previously with various experimental and computational methods.^59,69−71^ However, divalent cation electrolytes in acetonitrile have not been investigated, and no comprehensive account across the full concentration range from the dilute to the superconcentrated regime exists to the best of our knowledge.

In neat CD_3_CN, a single narrow C≡N stretch band has the asymmetric lineshape with a more pronounced absorption wing on the low-frequency side. Over the years, this asymmetry was ascribed to the formation of the acetonitrile dimer^72−75^ or a hot band^76−79^ caused by a small population of the lowest-frequency intramolecular mode (C–C≡N bend) at ∼369 cm^–1^ red-shifting the coupled C≡N stretch band by ∼5 cm^–1^. The hot band is the most likely explanation based on the self-consistent explanation of X-ray, neutron scattering, molecular simulation, matrix isolation gas-phase cluster, liquid relaxation, ab initio, and IR spectroscopic data.^80^

The 2262 cm^–1^ band intensity decreases upon the addition of salts, and a new band associated with the cation-coordinated C≡N stretch emerges at higher frequencies (Figure 2b, Supporting Information, Figure S4). The upshift of the nitrile stretch upon hydrogen bonding^19,20,80^ or ion coordination^38,79,81^ is well documented and can be qualitatively explained by the ion-induced shift of nitrile electron density toward the nitrogen that partially removes its contribution to the antibonding molecular orbital in the C≡N moiety, thus strengthening the bond. This polarization is reflected in the increase of the C≡N transition dipole moment (Figure S5). The spectral splitting between ion-coordinated and free C≡N bands in the low concentration limit (Δ) is also proportional to the strength of this ion–solvent interaction and increases with the charge density of the cation that reflects the polarizing ability of the metal (Figure 2c,d). Both splitting and transition dipole moments are related to the ion charge density and correlate strongly (Figure 2e). Weakly coordinating K^+^ does not induce spectral splitting, and its solubility is very low (Figure S3). Li^+^ ion leads to ∼25 cm^–1^ shift, which is slightly lower than the splitting it causes in organic carbonates (∼32 cm^–1^)^53^ in line with lower donicity of acetonitrile. Therefore, the strength of ion–acetonitrile interactions follows the expectations dictated by its Lewis basicity. Additionally, the ion–molecule interaction polarizes the nitrile. The band of the metal-coordinated C≡N stretch blue-shifts upon increase of salt concentration. This effect originates from the shrinking distance between the cation and the nitrogen atom and will be discussed in detail in the upcoming publication. At high electrolyte concentrations, the coordinated acetonitrile band dominates the spectrum, whereas the band of the free CD_3_CN strongly broadens but does not disappear completely. However, in superconcentrated solutions, there are as many or even more ions as solvent molecules, making the existence of free ACN, that is, ACN surrounded by other solvent molecules, highly unlikely. Therefore, at high concentrations, this band represents the acetonitrile located next to the TFSI^–^ anion but uncoordinated with the cation. In contrast to the strong cation–solvent interaction leading to ∼15–40 cm^–1^ upshift, the peak of the TFSI^–^ surrounded acetonitrile downshifts in frequency, and the small ∼1–2 cm^–1^ magnitude of the shift indicates the weak anion–solvent interaction. Even in the most extreme case of the superconcentrated Mg(TFSI)_2_, this band red-shifts by only ∼3.5 cm^–1^ (Figure S9). It conforms with the general notion that acetonitrile solvates negative ions poorly due to the lack of the localized electrophilic moiety.^48^ The broadening of the uncoordinated ACN band indicates a highly heterogeneous environment that solvent experiences around the anions in concentrated solutions. As a result, this spectral pattern allows for a clear distinction between various solvent configurations: cation-coordinated versus free ACN in the dilute regime and cation versus anion associated in concentrated electrolytes (Figure 2b). Overall, ACN demonstrates somewhat weaker ion–solvent interactions than classic Li-ion battery carbonates.

**Figure 3:**
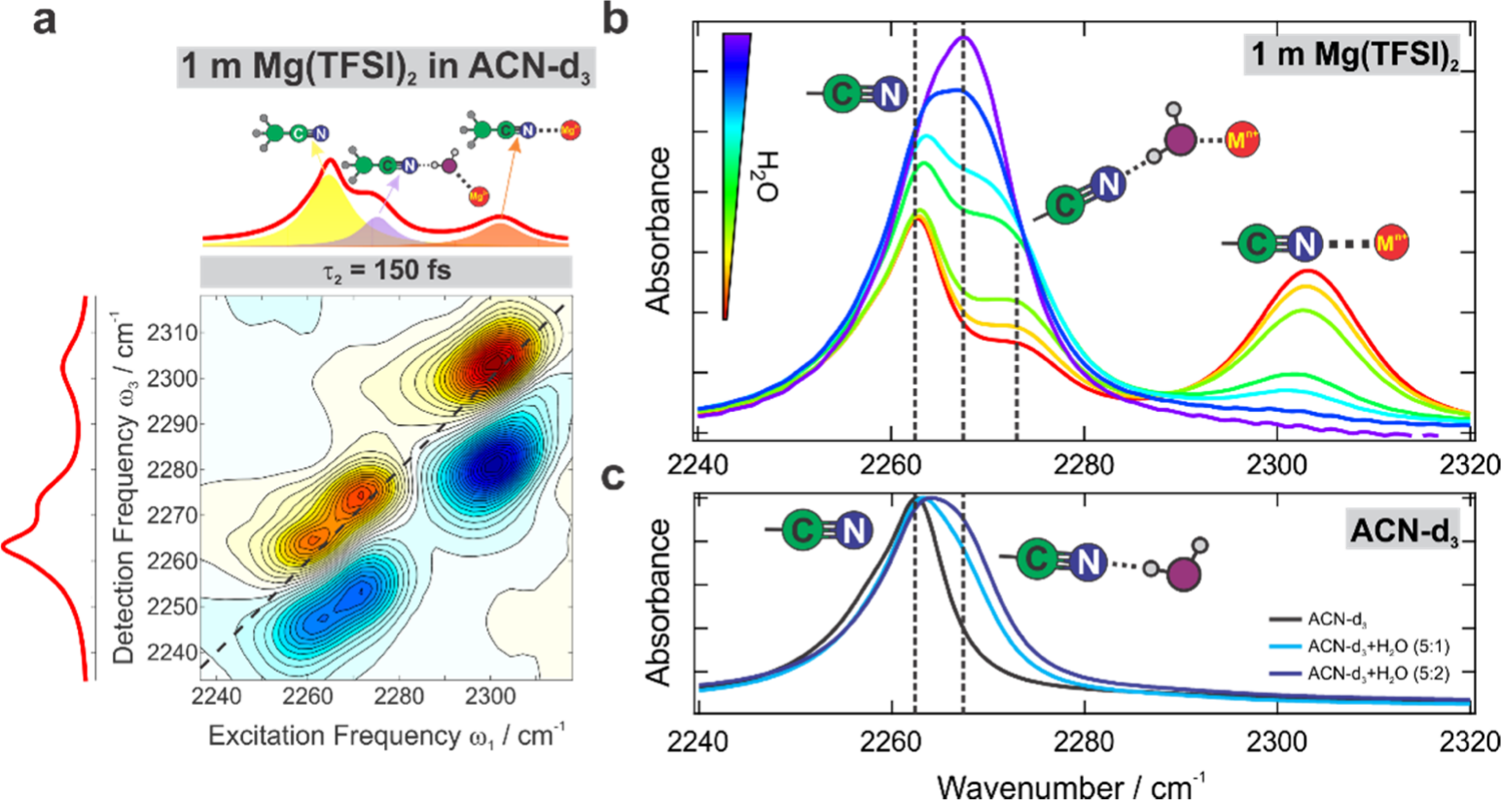
Influence of water on the C≡N stretch region in (a) 2D IR spectroscopy of the solvent in 1 m Mg(TFSI)_2_ electrolyte in CD_3_CN at early 150 fs waiting time. (b) Linear IR spectroscopy of 1 m Mg(TFSI)_2_ CD_3_CN solution upon addition of small amounts (∼1–10% v/v) of water. (c) Neat CD_3_CN upon addition of substantial amounts of water. Cartoons illustrate the species responsible for each peak.

Mg(TFSI)_2_ salt deserves some special attention. The addition of this salt to acetonitrile leads to an additional band at 2274 cm^–1^ (marked with an asterisk, Figures 2c and S4e) that grows proportionately with the coordinated nitrile at 2305 cm^–1^. Whereas the splitting between the latter band and the uncoordinated CD_3_CN at 2262 cm^–1^ is in line with the large charge density of the small double-charged Mg^2+^ ion (Figure 2d), the 2274 cm^–1^ band is at lower frequencies than the Ba^2+^-coordinated C≡N and is thus not representative of the high Mg^2+^ polarizing ability. The early-time 2D IR spectrum does not reveal cross-peaks between any of the three bands confirming that they originate from three distinct species (Figure 3a). The intensity of the 2274 cm^–1^ band critically depends on the water content growing significantly with the latter (Figure 3b). However, water alone in the absence of the Mg salt does not yield this band (Figure 3c) because the hydrogen-bonded acetonitrile upshifts by only several cm^–1^. Both a strongly polarizing cation and water are essential to yield this C≡N band. Therefore, we assign this band to the nitrile H-bonded to a water molecule that is itself coordinated to a Mg^2+^ cation. In other words, it is a second solvation shell acetonitrile molecule separated from the ion by a water molecule (Figure 3b). The strong Mg^2+^–water interaction involves substantial charge transfer between the ion and water molecules^82^ and causes significant enhancement of the H-bond donating ability of the water O–H bond that in turn causes the large shift of the IR resonance of the nitrile with which it forms a hydrogen bond.

The reason for the appearance of this species in nominally nonaqueous Mg(TFSI)_2_ electrolytes is that this salt is the most hygroscopic out of the ones used in the current study, and it carries a noticeable amount of water in its crystals that coordinates to the cation and produces this prominent peak in the IR spectrum. This result highlights that a strongly polarizing Mg^2+^ cation essentially acts as a desiccant in solution strongly attracting present water molecules, which outcompete acetonitrile for the place in the first solvation shell of the cation because of the higher donicity of water. This is in line with a recent investigation of aqueous mixed Zn(TFSI)_2_/LiTFSI electrolytes, where water is preferentially coordinated to the divalent Zn^2+^ in the presence of any quantities of competing Li^+^ cation or TFSI^–^ anion.^83^ This observation highlights the exquisite sensitivity of the acetonitrile C≡N stretch mode to variation in its environment associated with cations and their solvation structures. It also provides a useful means of detecting spurious water admixtures in nominally dry multivalent electrolyte salts.

### 3.2 Isotope Labeling Solvent

Although stationary IR spectroscopy of these electrolyte formulations clearly resolves multiple species present in the solution, it does not provide information on their dynamics. We aim to elucidate the dynamics of structural interconversions between multiple species with the help of 2D IR spectroscopy in the following study. To do that, we need to separate the dynamics of chemical exchange and vibrational energy transfer. A typical mitigation strategy, to prevent VET by keeping the vibrational reporter at low concentration, is obviously not possible in the experiments where a solvent is used as a vibrational probe. Therefore, we use an isotope-labeled acetonitrile (henceforth designated as label), whose C≡N stretch resonance frequency is shifted with respect to CD_3_CN but that is otherwise identical to the parent solvent. This strategy does not introduce additional additives in the solution beyond the species that are already present and thus avoids any perturbation of the solution morphology and dynamics.

A choice of the appropriate isotope-labeled acetonitrile is a crucial endeavor that determines the feasibility of the 2D IR chemical exchange experiments and the ease of the data interpretation. The purpose of the labeling is to make a C≡N stretch distinguishable from the uncoordinated CD_3_CN without interfering with the metal-coordinated CD_3_CN. Therefore, a typical labeling approach is to substitute the nitrile carbon for ^13^C, the nitrile nitrogen for ^15^N, or both. Two major considerations to be taken into account are the isotope-induced spectral shift and Fermi-resonance enhanced combination bands. We considered various isotopologues that can be broadly grouped into three families (Table 1). The protiated family consists of acetonitrile molecules that feature a labeled nitrile group attached to the protiated −CH_3_ methyl group. The deuterated family connects the labeled nitrile to the perdeuterated −CD_3_ methyl group. The 1-^13^C family can feature either type of hydrogen and is distinguished by ^13^C carbon in the methyl group.

**Table 1:** Acetonitrile Isotopologues Considered in This Work

protiated family	deuterated family	1-^13^C family
CH_3_CN	CD_3_CN^a^	^13^CH_3_^13^CN
CH_3_^13^CN	CD_3_^13^CN^a^	^13^CH_3_C^15^N
CH_3_C^15^N	CD_3_C^15^N	^13^CH_3_^13^C^15^N
CH_3_^13^C^15^N	CD_3_^13^C^15^N	^13^CD_3_^13^CN
		^13^CD_3_C^15^N
		^13^CD_3_^13^C^15^N

a Molecule of choice.

The main issue with the protiated family is the presence of the combination band of C–C stretch and C–H bend at frequencies higher than the C≡N stretch band that gains intensity from the latter via Fermi resonance (Figure 1a and Table S2).^64^ Accidental FR is a common situation in this spectral region^84^ and significantly complicates IR spectroscopy of the local modes. The key point is that the combination band not only borrows oscillator strength of the marker mode, thus effectively decreasing its intensity, but its location at higher frequencies and 30 cm^–1^ spectral separation from the C≡N band overlaps it significantly with the IR band of the cation-coordinated acetonitrile for most of the cations of interest (Figures 2c and S4). Anharmonic vibrational frequency analyses were carried out with density functional theory (DFT) calculations to estimate the isotope spectral shift and to determine if the other members of the protiated family suffer from the same issue. We found that the new position of the labeled C≡N stretch band effectively couples it with another C–C stretch + C–H bend combination band at lower frequencies (Figure 1b and Table S2). As a result, both CH_3_^13^CN and CH_3_^13^C^15^N suffer from the presence of the combination band at higher frequencies, whose intensity is even larger than in conventional CH_3_CN. It is worth noting that our anharmonic calculations performed at ωB97XD/6-311++G(d,p) level of theory as implemented in Gaussian 16^85^ do not capture the Fermi coupling (for example, CH_3_CN and CD_3_CN IR bands are predicted at identical frequencies with no combination band at higher frequencies for the former) and therefore underestimate the vibrational coupling. For this reason, CH_3_C^15^N that has the lowest isotope shift (−24 cm^–1^), and whose combination band appears at lower frequencies (2205 cm^–1^), is still expected to suffer from FR to the C–C stretch + C–H bend combination band at higher frequencies (this is supported by the results from the 1-^13^C family presented below). Additionally, such a low isotope shift makes it unsuitable because the cation-coordinated CH_3_C^15^N will overlap with the uncoordinated CD_3_CN at 2262 cm^–1^. As a result, none of the members of the protiated family can usefully serve as a label in acetonitrile electrolytes.

The deuterated family does not suffer from the abovementioned combination band because the C–D bending mode appears at much lower frequencies than C–H bends. This makes the C≡N stretch band of CD_3_CN appear as a single sharp band in this spectral region with no notable combination bands (Figure 1a). The 10 cm^–1^ difference in the C≡N peak position between CD_3_CN and CH_3_CN is exactly due to the FR in the latter. As will become clear below, CD_3_CN is in the sweet spot where no FR is present in contrast to all other isotopologues. Isotope labeling the nitrile group in deuterated acetonitrile shifts it to lower frequencies where it couples to the C–D stretch or the C–D bend overtone. Figure 1a demonstrates the experimental IR spectrum of CD_3_^13^CN, where a notable two-peak structure (2198 and 2224 cm^–1^) is evident. In the following subsections, we argue that this structure originates from the interaction of the bright C≡N stretch and the dark C–D stretch transitions. This coupling has several disadvantages: it diminishes the intensity of the C≡N marker mode by a factor of >2 and shifts its peak to higher frequencies compared to where it would appear in the absence of the FR, thus effectively hampering our labeling strategy. However, the most important point is that the coupling dark mode appears at lower frequencies relative to the ^13^C≡N marker mode and does not interfere with the cation-coordinated band at higher frequencies with respect to the free ^13^C≡N. There is another very weak band at 2251 cm^–1^ of unassigned origin that goes away upon the addition of the salt and thus does not play a role in our experiments.

CD_3_C^15^N is not a good option because its isotope shift (−24 cm^–1^) is too small, making its ion-coordinated band overlap with CD_3_CN in a similar way as described for the protiated family (Figure 1b and Table S2). Oppositely, CD_3_^13^C^15^N is expected to have an isotope shift that is too large (−77 cm^–1^) placing it at ∼2185 cm^–1^, where it will additionally enter into FR with the overtone of the C–D bending mode at 2198 cm^–1^, which is again at higher frequencies with respect to the C≡N stretch making it unsuitable for ion-coordination experiments.

If the deuterated family represents an attempt to circumvent the C–C stretch + C–H bend combination band issue encountered with the protiated family by shifting the frequency of the C–H bending mode, the 1-^13^C family is an attempt to get away from it by shifting the frequency of the C–C stretch band instead. Therefore, a label with two ^13^C carbons should provide the best opportunity for that. Figure 1a shows a spectrum of ^13^CH_3_^13^CN. Clearly, the detuning is not large enough to fully suppress the FR with this combination band, as it is clearly observed at 2260 cm^–1^. Moreover, this combination band precisely overlaps with the C≡N stretch band of CD_3_CN—the main acetonitrile isotopologue in our experiments—thus precluding their utilization in the mixture. ^13^CH_3_C^15^N would not work either due to the even smaller isotope shift of the C≡N band. Possibly, ^13^CH_3_^13^C^15^N might be the best option because its isotope shift is so large that the FR should be fully suppressed, and it does not contain C–D bends. However, triple isotope labeling of such a light volatile molecule to be produced in quantities sufficient for use as a solvent is an expensive undertaking that we have not attempted. From Figure 1a, it is clear that ^13^CD_3_^13^CN is not a useful alternative because the C≡N stretch at 2202 cm^–1^ almost precisely overlaps with the overtone of the C–D bending mode at 2198 cm^–1^ in addition to the C–D stretch mode at 2214 cm^–1^ that contributes in CD_3_^13^CN discussed above (see also the following subsection). Therefore, multiple strong Fermi resonances are expected. In the case of ^13^CD_3_C^15^N, the smaller isotope shift and overlap with the C–D stretch should make its spectrum similar to that of CD_3_^13^CN, which is a much simpler and cost-effective option. ^13^CD_3_^13^C^15^N as a fully isotope-substituted version of acetonitrile solvent is definitely an interesting option to consider for spectroscopy, but its cost is prohibitively high.

In the end, even an extensive search for an isotope-edited label (Table 1) may not guarantee complete avoidance of accidental Fermi resonances.^84,86^ All of these considerations highlight that CD_3_CN is the only acetonitrile isotopologue whose C≡N stretch does not suffer from intensity borrowings and Fermi resonances of nearby dark modes, and CD_3_^13^CN is not ideal but the most feasible isotope-labeled solvent for ion-coordinating experiments to be employed in mixture with CD_3_CN.

### 3.3 Fermi Resonance in the Label

**Figure 4:**
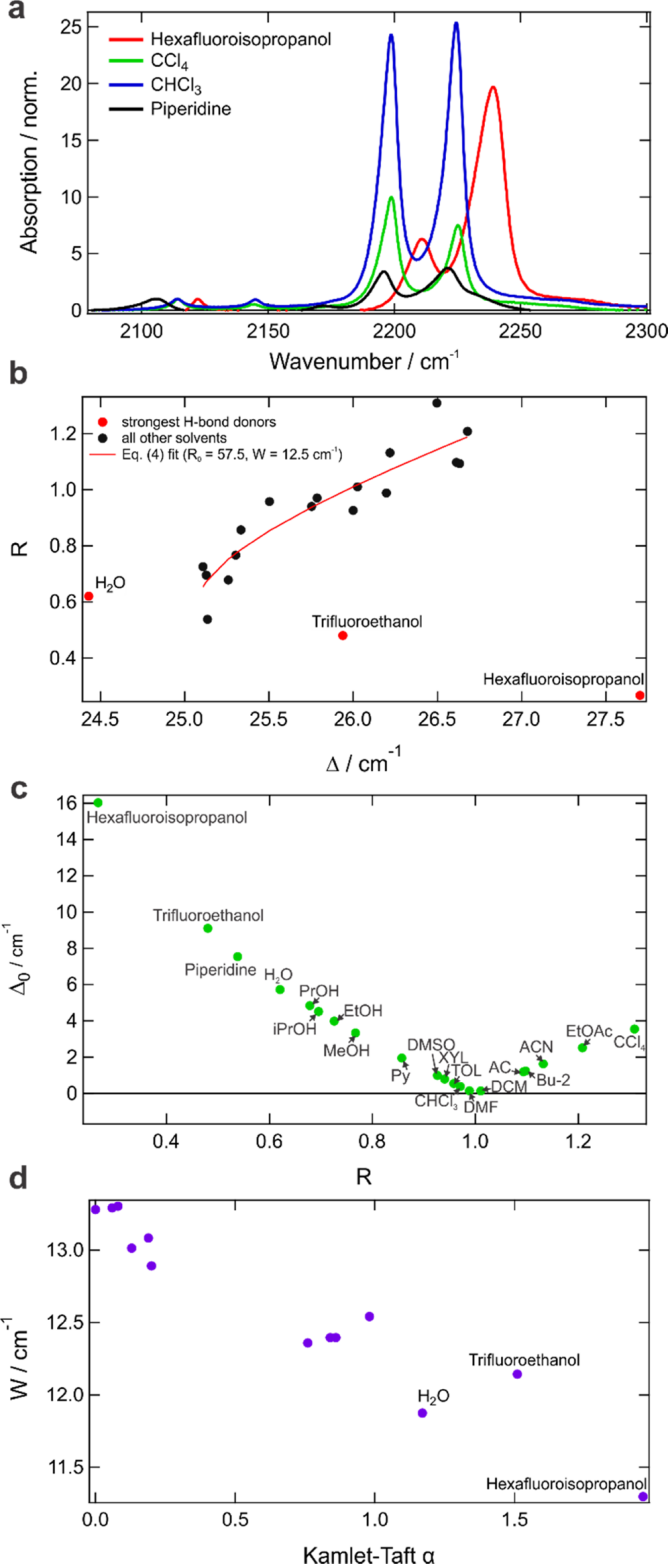
Fermi resonance in the label. (a) Exemplary IR spectra of the 3% solution of the CD_3_^13^CN label in the C≡N stretch region in various solvents. All spectra are normalized to the C–D symmetric stretch band at 2112–2122 cm^–1^. (b) *R* as a function of Δ for all solvents; the three strongest H-bond donating ones are marked in red and labeled. The fit according to eq 4 is applied to all solvents except for the three outliers and is shown as a solid line. (c) Dependence of Δ_0_ on *R* for all studied solvents. Nonstandard solvent abbreviations: Py (pyridine), XYL (xylene), TOL (toluene), AC (acetone), and Bu-2 (butanone-2). (d) Fermi coupling constant, *W*, as a function of solvent H-bond donating ability α.

**Figure 5:**
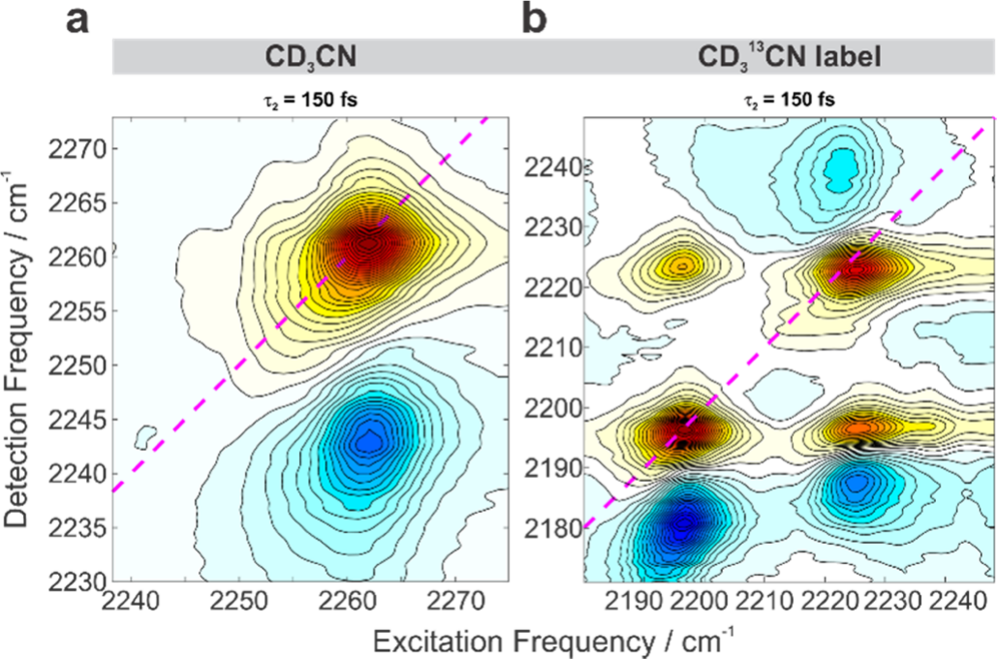
Comparison of the 2D IR C≡N stretch spectroscopy in CD_3_CN and the CD_3_^13^CN label: (a) neat CD_3_CN at early 150 fs waiting time. (b) Neat CD_3_^13^CN label at early 150 fs waiting time. Ground-state bleaches are shown in red and excited-state absorptions in blue. The diagonal is a dashed magenta line.

The CD_3_^13^CN label features Fermi resonance between the C≡N stretch mode and a dark mode that borrows intensity from it (Figure 1a). We established the presence of Fermi resonance via the method of solvent variation^87^ and with 2D IR spectroscopy^88^ (Figures 4 and 5). The IR spectrum of the label was measured in 20 solvents, whose differing polarity and hydrogen-bond donating ability induce spectral shifts of the nitrile band that perturb the FR with the dark mode (Figure S6).

Figure 4a illustrates how a change of solvent results in a change in the splitting (Δ) and intensity ratio (*R*) of the two Fermi-coupled bands. These variables are defined as
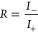
1
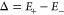
2where “–” refers to the lower and “+” to the higher frequency coupled bands and are shown in Figure 4b for all solvents. Figure 4c shows a typical V-shape dependence of the energy difference between the unperturbed energy levels (that is, in the absence of Fermi resonance), Δ_0_, on *R*. It visualizes a common notion that at the degeneracy point, the Fermi coupling brings both bands to the same intensity, whereas their intensity ratio becomes more disparate with frequency detuning. Δ_0_ can be determined from
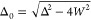
3where *W* is the Fermi coupling constant. The Fermi coupling itself can be determined from the experimental observables Δ and *R* via the following relation
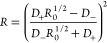
4where *R*_0_ is the ratio of the band intensities in the absence of FR and
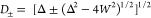
5It implies that if the coupling constant remains the same within a solvent set, both *R*_0_ and *W* can be obtained by fitting eq 4 to the experimental data. Figure 4b shows that for the vast majority of solvents this is indeed the case as all of them fall on the same curve, except for the three with the highest H-bond donating ability (water, trifluoroethanol, and hexafluoroisopropanol). The solid line shows the best fit of eq 4 that corresponds to the Fermi coupling constant *W* = 12.5 cm^–1^ and the intensity ratio of the Fermi-decoupled bands *R*_0_ = 57.5. Such a large value of *R*_0_ shows that the intensity of the dark mode is less than 2% of the bright one in the absence of the FR and justifies a common simplification assuming *R*_0_ = ∞, that is, the unperturbed transition is essentially forbidden. In that case, one obtains
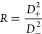
6From eqs 5 and 6, the coupling constant can be determined for all solvents and its variation can be examined. The variation of the Fermi coupling constant is small in accord with the results obtained from the full fit to eq 4. For the majority of solvents, *W* = 12.5–13.0 cm^–1^ and does not depend on solvent polarity (Figure S7) but depends on the hydrogen bond donating ability, showing a linear decrease with the increase of the latter as quantified by, e.g., Kamlet–Taft α parameter (Figure 4d).^89^ It explains why the three most potent H-bond donating solvents do not follow the general trend presented in Figure 4b. This magnitude of Fermi coupling constant is slightly lower than that in neat conventional CH_3_CN (*W* = 19.6 cm^–1^).^64^

Early waiting time (τ_2_ = 150 fs) 2D IR spectra provide another evidence of the Fermi resonance (Figure 5). Whereas in neat CD_3_CN, there is only a single excited C≡N stretch transition manifested as a single bleach on the diagonal and an excited-state absorption band anharmonically downshifted along the probe (detection) axis (Figure 5a), a 2D IR spectrum of the label is more peculiar (Figure 5b). At the earliest waiting time, both diagonal bleaches are present at 2198 and 2224 cm^–1^, and their corresponding excited-state absorption bands are located, respectively, at lower (2180 cm^–1^) and higher (2240 cm^–1^) probe frequencies. This unusual pattern of the excited-state absorption being at higher frequency than the diagonal is a typical manifestation of Fermi coupling in agreement with the previous theoretical and 2D IR investigations of FR.^88,90^ The cross-peak bleaches feature a half of the intensity of the diagonal ones, and the upper left cross-peak is located at (ω_1_, ω_3_) = (2198, 2224 cm^–1^) does not have an accompanying excited-state absorption band.^88^

### 3.4 Nature of the Fermi Coupled Dark Mode

**Figure 6:**
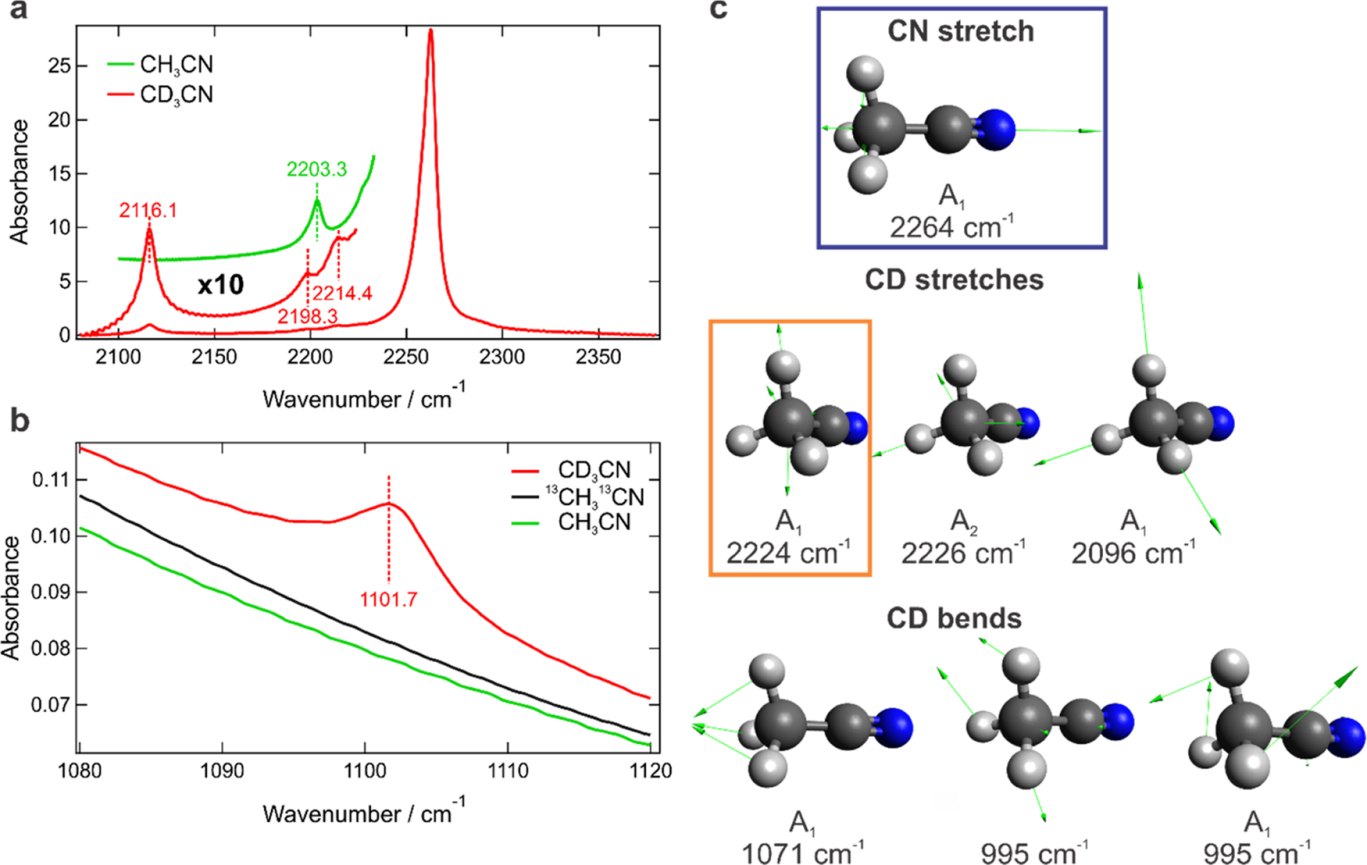
Nature of the coupled dark mode. (a) IR spectrum of CD_3_CN normalized to the absorption band of the totally symmetric C–D stretch mode around 2115 cm^–1^. The inset shows a 10× magnification of the low-intensity part of the CD_3_CN (red) and CH_3_CN (green) spectra. The CH_3_CN spectrum is offset for clarity. (b) Totally symmetric C–D bend region in CD_3_CN compared to ^13^CH_3_^13^CN and CH_3_CN, confirming that this band originates from the C–D bending mode. Apparent peak maxima of low-intensity bands are marked in panels (a,b). (c) Calculated frequencies and displacement vectors of the relevant vibrational modes in CD_3_CN. Emerging C–D stretch around 2224 cm^–1^ (orange frame) is the most likely candidate to borrow intensity from the C≡N stretch (indigo frame) in the labeled molecule. Anharmonic calculations were performed at ωB97XD/6-311++G(d,p) level of theory with the PCM solvent model of acetonitrile. The scaling factor of 0.949 was applied according to ref (96).

Now we turn to the nature of the mode entering into Fermi resonance with the C≡N stretch. Typically for FR, a dark mode is a combination band or an overtone. However, in this case, the most likely candidate is a dark C–D stretch fundamental mode whose frequency is predicted by DFT calculations to appear around 2224 cm^–1^ (Figure 6c), in good agreement with our estimation of the degeneracy point of the unperturbed bands near 2213 cm^–1^ from the experimental data using eqs 1–6. It is worth noting that it is not a totally symmetric C–D stretch that has the appropriate A_1_ symmetry and consistently appears as a weak band around 2115 cm^–1^ (Figures 4, 6, and S6) (calculated at 2096 cm^–1^, Figure 6c) but another C–D stretch band that can only arise from the vibrations of slightly distorted acetonitrile molecules of non-*C*_3*v*_ symmetry (as there can be only one A_1_-type C–D stretch mode in the *C*_3*v*_ group). Our calculations indicate that even the slightest distortions of the C–D–C angle cause the appearance of this band around 2224 cm^–1^. It is not surprising that such symmetry breaking can be present in highly polar solvents like acetonitrile where strong fluctuations of electric fields and solvent motion can cause significant molecular asymmetry.^91^ It is possible to observe this extremely weak but clearly discernible band in the absence of the FR in CD_3_CN peaking at 2214.4 cm^–1^ (Figure 6a). The absence of this weak band in protiated CH_3_CN (Figure 6a) provides additional evidence that the Fermi resonance is associated with C–D vibrations. The band at 2203.3 cm^–1^ in CH_3_CN is associated with the C≡N stretch of the CH_3_^13^CN isotopologue present due to the natural ∼1.1% abundance of ^13^C as confirmed by: (i) the relative integral intensity of this band with respect to the integral C≡N stretch intensity (1.5%); (ii) the absence of any bands at half the energy (green line, Figure 6b) excluding the assignment of this band to an overtone; and (iii) close correspondence to the calculated 2198 cm^–1^ frequency of the C≡N stretch in this isotopologue (Figure 1b).

In contrast, the C–D bend overtone is not at the origin of the Fermi resonance. The totally symmetric C–D bend mode fundamental transition appears at 1101.7 cm^–1^ (Figure 6b) (calculated at 1071 cm^–1^, Figure 6c) in accord with the previous observations,^92^ and therefore, the weak band at 2198.3 cm^–1^ (Figure 6a) can be assigned to its overtone in agreement with the expected weak anharmonicity typical for intramolecular vibrations. We emphasize that the band at 2214.4 cm^–1^ cannot represent its overtone because that would imply the negative anharmonicity of this mode, the phenomenon that is observed extremely rarely for molecular vibrations originating from the quantum confinement^93−95^ and that has no justification in this case. No other combination bands or overtones were found near the point of degeneracy, and therefore, we conclude that the C–D stretch fundamental vibration arising from the symmetry breaking in polar acetonitrile is the most likely candidate responsible for the interaction with the C≡N stretch in the CD_3_^13^CN label. Therefore, this interaction should be broadly classified as intensity borrowing rather than Fermi resonance, which is associated with the cubic force constant that couples an optically bright mode with the dark overtone or the combination band instead of a fundamental transition.

### 3.5 Interaction of the Label with Salts

**Figure 7:**
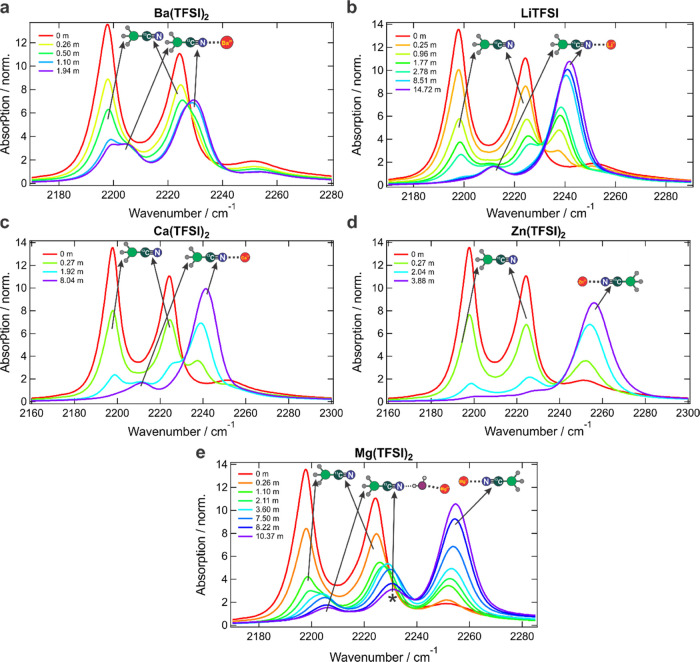
IR spectra in the solvent C≡N stretch region of the CD_3_^13^CN label upon addition of various amounts of M(TFSI)*_n_* salts of increasing cation charge density: Ba^2+^ (a), Li^+^ (b), Ca^2+^ (c), Zn^2+^ (d), and Mg^2+^ (e). The asterisk denotes the nitrile H-bonded to metal-coordinated water. All spectra are normalized to the absorption band of the symmetric C–D stretch mode at 2115 cm^–1^. Band assignments are indicated with arrows and cartoons.

The addition of M(TFSI)*_n_* salts to the label causes changes in the nitrile region of its IR spectrum similar to CD_3_CN described earlier (Section 3.1): a new band of the metal-coordinated C≡N stretch appears at higher frequencies determined by the cation charge density (Figure 7). However, due to the intensity-borrowing interaction with the C–D stretch, the spectral pattern is more complex. The C≡N frequency upshift leads to the perturbation of the FR that is manifested as a corresponding upshift of the C–D stretch band to 2205–2210 cm^–1^ and its intensity loss (Figure 7a–c). Performing the analysis described in Section 3.4, we found that the coupling constant in the metal-coordinated label decreases compared to the free solvent. This decrease is related to the metal-ion charge density. For example, while *W* = 13 cm^–1^ in free CD_3_^13^CN, it reduces to *W* = 10 cm^–1^ in Ba^2+^-coordinated species, *W* = 9 cm^–1^ for Ca^2+^-coordinated ACN, and *W* = 8 cm^–1^ in Li^+^-bound CD_3_^13^CN. This decrease in the coupling upon interaction with metal ions is consistent with the previously reported decrease of *W* for Li^+^-coordinated conventional CH_3_CN compared to the free CD_3_^13^CN (*W* = 14.3 vs 19.6 cm^–1^, respectively).^64^ Furthermore, in our case, the most polarizing and hence the most perturbing Zn^2+^ and Mg^2+^ cations disrupt the FR completely for the cation-coordinated solvent. This is obvious in Zn^2+^ electrolytes that demonstrate only a single peak of the cation-coordinated C≡N stretch around 2256 cm^–1^ in the presence of high quantities of Zn^2+^ salt (Figure 7d). In the case of Mg^2+^, the situation is complicated by the presence of the water admixture that causes the appearance of the nitrile H-bonded to metal-coordinated water (Figure 3b), yielding an unusual three-peak structure (Figure 7e). This spectral pattern originates from the superposition of a single metal-coordinated nitrile band around 2255 cm^–1^ (similar to the Zn^2+^ case: a single band, no intensity borrowing), and the water-mediated species whose C≡N stretch peak appears around 2230 cm^–1^ (Figure 7e, marked with an asterisk) and its corresponding coupled C–D stretch companion is at 2206 cm^–1^. This pattern is understood in detail with the help of 2D IR spectroscopy and is explicated in Supporting Information, Section S2.4. Overall, like in the case of a free solvent, the intensity-borrowing resonance causes a more complex spectral pattern in the C≡N stretch region upon metal coordination and reduces the oscillator strength of the coordinated C≡N stretch band. However, it still can be used in a similar way as CD_3_CN to track local ion–solvent interactions since the splitting between the coordinated and noncoordinated solvent bands is sufficient to differentiate them.

## 4 Conclusions

Utilization of intrinsic vibrational reporters naturally present in electrolytes without resorting to the extrinsic molecular probes is the most reliable way of extracting the relevant information about the nature of these liquids, especially in unconventional concentrated and superconcentrated regimes. Previously, we have used the vibrational normal modes of the TFSI^–^ anion to interrogate ion–ion interactions and networking in aqueous LiTFSI formulations across the concentration range.^97^ Here, we have demonstrated that the solvent local C≡N stretch of acetonitrile is a useful intrinsic vibrational marker to monitor ion–solvent interactions in various novel nonaqueous electrolyte formulations: from the classic Li^+^ to the novel divalent salt formulations based on Mg^2+^, Ca^2+^, and Zn^2+^ charge carriers. It is most sensitive to cation–solvent interactions responding by the appearance of a distinct ion-coordinated C≡N band whose transition dipole moment and spectral position are proportional to the strength of the ion–solvent interaction, which is in turn determined by the charge density of the cation and donicity of the solvent. The water, which can be present as an admixture in the most hygroscopic divalent salts, outcompetes acetonitrile for direct coordination with the cations. The acetonitrile is displaced into the second solvation shell of the cation and accepts a hydrogen bond from the metal-coordinated water. Due to the strong ion–water interaction that polarizes the O–H moiety, this hydrogen bond is significantly stronger than a typical water–nitrile H-bond in the absence of metal ions and shifts the C≡N stretch resonance to a distinct position in the IR spectrum that is different from both the free and directly metal-coordinated species. This peculiar spectroscopic feature can be useful to detect the presence of spurious water content that has a deleterious effect on the electrochemical properties of the system in nominally anhydrous electrolyte salts.^98^

Out of numerous acetonitrile isotopologues, CD_3_CN is the best candidate for a vibrational probe as it features a single, narrow intense band in the C≡N stretch IR region and does not suffer from intensity-borrowing couplings from nearby dark modes. However, to discern the dynamics of structural interconversions among various species present in liquid electrolytes from the vibrational energy-transfer processes, the need emerges for the isotopically edited acetonitrile featuring a spectroscopically distinct C≡N stretch that could be differentiated from the CD_3_CN isotopologue in the presence of various salts. We demonstrated that CD_3_^13^CN appears the most feasible option to function as such an intrinsic label. It is not as ideal as CD_3_CN since it suffers from the intensity borrowing that couples the C≡N and C–D stretches and effectively reduces the intensity of the C≡N mode and complicates the IR spectroscopy of these liquids. Nevertheless, the spectral picture remains tractable, and the most important advantage of using the solvent mode to interrogate structural dynamics in these liquids is that it necessarily provides a full picture of ion–solvent interactions and does not suffer from the perturbations imposed by the introduction of extrinsic molecular probes. In the future publication, we will report how using a mixture of these two acetonitrile solvent isotopologues allowed us to separate 2D IR spectral dynamics of various ion solvation structures into the chemical exchange and energy-transfer components—the information critical for the assessment of the charge transport mechanisms in novel battery electrolytes.

## Supporting Information Available

Solution compositions, synthesis of the vibrational label, additional spectroscopic data, supplementary references, Figures S1–S11, and Tables S1 and S2 (PDF)
