# Supplementary material for: Characterization of Acetonitrile Isotopologues as Vibrational Probes of Electrolytes
Source: J Phys Chem B. 2021 Dec 28;126(1):278–91. doi: 10.1021/acs.jpcb.1c09572 (PMC8762666; doi:10.1021/acs.jpcb.1c09572)
Supplement: Supplementary file 1 — jp1c09572_si_001.pdf [file jp1c09572_si_001.pdf]

SUPPORTING INFORMATION

Characterization of Acetonitrile Isotopologues as Vibrational Probes of Electrolytes

Bogdan Dereka,[1,2,3,4] Nicholas H. C. Lewis,[1,2,3,4] Jonathan H. Keim,[2] Scott A. Snyder,[2] and Andrei Tokmakoff[1,2,3,4]*

[1]*James Franck Institute, The University of Chicago, Chicago, Illinois 60637, USA*

[2] *Department of Chemistry, The University of Chicago, Chicago, Illinois 60637, USA*

[3]*Institute for Biophysical Dynamics, The University of Chicago, Chicago, Illinois 60637, USA*

[4]*Joint Center for Energy Storage Research, Argonne National Laboratory, Lemont, Illinois 60637, USA*

* Corresponding author, e-mail: tokmakoff@uchicago.edu

Table of Contents

Table of Contents S1

S1 EXPERIMENTAL DETAILS S1

  S1.1 Solution Compositions S1

  S1.2 Synthesis of the Label S3

S2 ADDITIONAL SPECTROSCOPIC DATA S5

  S2.1 Linear IR Spectra of C≡N Stretch upon Interaction with Salts S5

  S2.2 Fermi Resonance in the Label S8

  S2.3 Vibrational modes of various acetonitrile isotopologues in the C≡N stretch region S9

  S2.4 Influence of Water on IR Spectra of Solvent C≡N Stretch S10

S3 SUPPLEMENTARY REFERENCES S14

S1 EXPERIMENTAL DETAILS

S1.1 Solution Compositions

Table S1. Solution compositions for electrolyte formulations used in the current study (ω denotes mass fraction, x denotes molar fraction).

LiTFSI in CD_3CN							
Molality / m	Molarity / M	Solvent Molarity / M	Solvent molecules per ion	ω (solute)	ω (solvent)	x (solute)	x (solvent)
0.2587	0.2151	18.8708	43.8562	0.0691	0.9309	0.0113	0.9887
0.9939	0.7271	16.5999	11.4152	0.2220	0.7780	0.0420	0.9580
2.0940	1.3215	14.3205	5.4181	0.3755	0.6245	0.0845	0.9155
3.9943	2.2025	12.5119	2.8404	0.5342	0.4658	0.1497	0.8503
6.1006	2.8862	10.7352	1.8597	0.6366	0.3634	0.2119	0.7881
8.7000	3.5619	9.2901	1.3041	0.7141	0.2859	0.2771	0.7229
12.006	4.4360	8.3839	0.9450	0.7751	0.2249	0.3460	0.6540
15.893	5.4027	7.7137	0.7139	0.8202	0.1798	0.4119	0.5881
LiTFSI in label							
Molality / m	Molarity / M	Solvent Molarity / M	Solvent molecules per ion	ω (solute)	ω (solvent)	x (solute)	x (solvent)
0.2502	0.2050	18.5919	45.3461	0.0670	0.9330	0.0109	0.9891
0.9616	0.7019	16.5641	11.7987	0.2163	0.7837	0.0407	0.9593
1.7700	1.1742	15.0532	6.4099	0.3369	0.6631	0.0724	0.9276
2.7759	1.6741	13.6847	4.0872	0.4435	0.5565	0.1090	0.8910
8.5080	3.5465	9.4586	1.3335	0.7095	0.2905	0.2727	0.7273
10.634	4.0409	8.6226	1.0669	0.7533	0.2467	0.3191	0.6809
14.718	5.0984	7.8603	0.7709	0.8086	0.1914	0.3934	0.6066
$Mg(TFSI)_2$ in CD_3CN							
Molality / m	Molarity / M	Solvent Molarity / M	Solvent molecules per ion	ω (solute)	ω (solvent)	x (solute)	x (solvent)
0.2671	0.1956	16.6198	28.3179	0.1351	0.8649	0.0116	0.9884
1.2031	0.7097	13.3856	6.2869	0.4129	0.5871	0.0504	0.9496
2.4746	1.1729	10.7555	3.0565	0.5913	0.4087	0.0983	0.9017
3.9624	1.5299	8.7613	1.9089	0.6985	0.3015	0.1487	0.8513
8.6173	2.0009	5.2687	0.8777	0.8344	0.1656	0.2752	0.7248
10.5049	2.0889	4.5121	0.7200	0.8600	0.1400	0.3165	0.6835
$Mg(TFSI)_2$ in label							
Molality / m	Molarity / M	Solvent Molarity / M	Solvent molecules per ion	ω (solute)	ω (solvent)	x (solute)	x (solvent)
0.2556	0.1879	16.6834	29.5920	0.1300	0.8700	0.0111	0.9889
1.1046	0.6647	13.6554	6.8475	0.3924	0.6076	0.0464	0.9536
2.1058	1.0664	11.4908	3.5919	0.5518	0.4482	0.0849	0.9151
3.6000	1.4508	9.1443	2.1010	0.6779	0.3221	0.1369	0.8631
7.5000	1.9161	5.7970	1.0085	0.8143	0.1857	0.2484	0.7516
8.2231	1.9755	5.4512	0.9198	0.8278	0.1722	0.2660	0.7340
10.3668	2.0850	4.5637	0.7296	0.8584	0.1416	0.3136	0.6864
$Ca(TFSI)_2$ in CD_3CN							
Molality / m	Molarity / M	Solvent Molarity / M	Solvent molecules per ion	ω (solute)	ω (solvent)	x (solute)	x (solvent)
0.2747	0.2025	16.7283	27.5345	0.1416	0.8584	0.0120	0.9880

0.9067	0.594	14.8649	8.3420	0.3525	0.6475	0.0384	0.9616
1.9388	1.0594	12.3994	3.9012	0.5379	0.4621	0.0787	0.9213
3.7933	1.5813	9.4590	1.9940	0.6949	0.3051	0.1432	0.8568
8.1210	2.0871	5.8315	0.9314	0.8298	0.1702	0.2636	0.7364
**$Ca(TFSI)_2$ in label**							
**Molality / m**	**Molarity / M**	**Solvent Molarity / M**	**Solvent molecules per ion**	**ω (solute)**	**ω (solvent)**	**x (solute)**	**x (solvent)**
0.2732	0.2015	16.7354	27.6857	0.1409	0.8591	0.0119	0.9881
1.9169	1.0519	12.4520	3.9458	0.5351	0.4649	0.0779	0.9221
8.0352	2.0695	5.8441	0.9413	0.8283	0.1717	0.2615	0.7385
**$Zn(TFSI)_2$ in CD_3CN**							
**Molality / m**	**Molarity / M**	**Solvent Molarity / M**	**Solvent molecules per ion**	**ω (solute)**	**ω (solvent)**	**x (solute)**	**x (solvent)**
0.2541	0.1999	17.8517	29.7667	0.1372	0.8628	0.0111	0.9889
1.045	0.6858	14.8907	7.2380	0.3953	0.6047	0.0440	0.9560
2.0558	1.1645	12.8528	3.6792	0.5626	0.4374	0.0831	0.9169
4.2164	1.7616	9.4803	1.7939	0.7251	0.2749	0.1567	0.8433
**$Zn(TFSI)_2$ in label**							
**Molality / m**	**Molarity / M**	**Solvent Molarity / M**	**Solvent molecules per ion**	**ω (solute)**	**ω (solvent)**	**x (solute)**	**x (solvent)**
0.2728	0.2134	17.7527	27.7263	0.1458	0.8542	0.0119	0.9881
2.0421	1.1566	12.8514	3.7039	0.5610	0.4390	0.0826	0.9174
3.8830	1.6417	9.5938	1.9479	0.7084	0.2916	0.1461	0.8539
**$Ba(TFSI)_2$ in CD_3CN**							
**Molality / m**	**Molarity / M**	**Solvent Molarity / M**	**Solvent molecules per ion**	**ω (solute)**	**ω (solvent)**	**x (solute)**	**x (solvent)**
0.2621	0.1974	17.0925	28.8582	0.1546	0.8454	0.0114	0.9886
1.0821	0.6832	14.3254	6.9899	0.4302	0.5698	0.0455	0.9545
2.1404	1.0977	11.6369	3.5338	0.5989	0.4011	0.0862	0.9138
4.1451	1.4912	8.1629	1.8247	0.7430	0.2570	0.1545	0.8455
6.2388	1.6902	6.1473	1.2124	0.8132	0.1868	0.2157	0.7843
**$Ba(TFSI)_2$ in label**							
**Molality / m**	**Molarity / M**	**Solvent Molarity / M**	**Solvent molecules per ion**	**ω (solute)**	**ω (solvent)**	**x (solute)**	**x (solvent)**
0.2605	0.1963	17.1029	29.0354	0.1538	0.8462	0.0113	0.9887
0.5000	0.3536	16.0476	15.1274	0.2586	0.7414	0.0216	0.9784
1.1000	0.6914	14.2614	6.8761	0.4342	0.5658	0.0462	0.9538
1.9370	1.0263	12.0226	3.9049	0.5747	0.4253	0.0786	0.9214

S1.2 Synthesis of the Label

$K^{13}CN$ (99%, C-13) was purchased from Cambridge Isotope Laboratories. CD_3I (99.5%, D) and triethylene glycol (anhydrous, BioUltra grade) were purchased from Sigma Aldrich. All chemicals were used as

received without further purification. The ^{2}H NMR spectra were recorded at 76 MHz and ^{13}C NMR spectra were recorded at 125 MHz on a Bruker 500 MHz spectrometer. The following abbreviations are used to explain the multiplicities: s = singlet, d = doublet. High-resolution mass spectra (HRMS) were recorded on an Agilent 7200B Q-Tof GCMS.

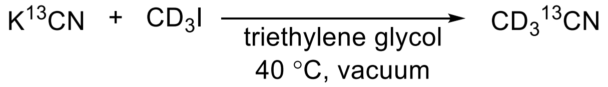

Using a modified procedure from Ref. 1, an oven-dried Schlenk flask with a magnetic stir bar was attached to a vacuum gas manifold and flushed with N_2. $K^{13}CN$ (4.96 g, 75 mmol, 1.2 eq), powdered in a mortar with a pestle, was added to the flask followed by triethylene glycol (8.9 mL, 7 M). The flask was then cooled to 0˚C and CD_3I (3.9 mL, 62.5 mmol, 1 eq) was added dropwise to the suspension (**Warning:** Exotherm upon addition). Upon completion of the addition, the reaction was cooled in a liquid N_2 bath until completely frozen. The flask was then evacuated, sealed, and heated to 40˚C for 12 hr. The reaction was then cooled to 23˚C. The product was isolated via distillation using a vacuum line (0.1 mm Hg) fitted with two vacuum traps, the first cooled to – 30˚C and the second cooled to – 90˚C to collect the desired $CD_3{}^{13}CN$ (2.06 g, 45.6 mmol, 73%) as a pale-yellow liquid containing approximately 3 mol% CD_3I as a contaminant. ^{2}H NMR ($CHCl_3$, 76 MHz) δ 1.46 (d, J = 1.5 Hz, 3 D). ^{13}C NMR ($CDCl_3$, 100 MHz) δ 116.5 (s, ^{13}CN), the CD_3 signal was not observed. HRMS (EI) calcd for $CD_3{}^{13}CN$ $[M]^+$ 45.0487, found 45.0486. The isotopic purity was determined to be >95% using the method as in ref. [2].

$CD_3{}^{13}CN$

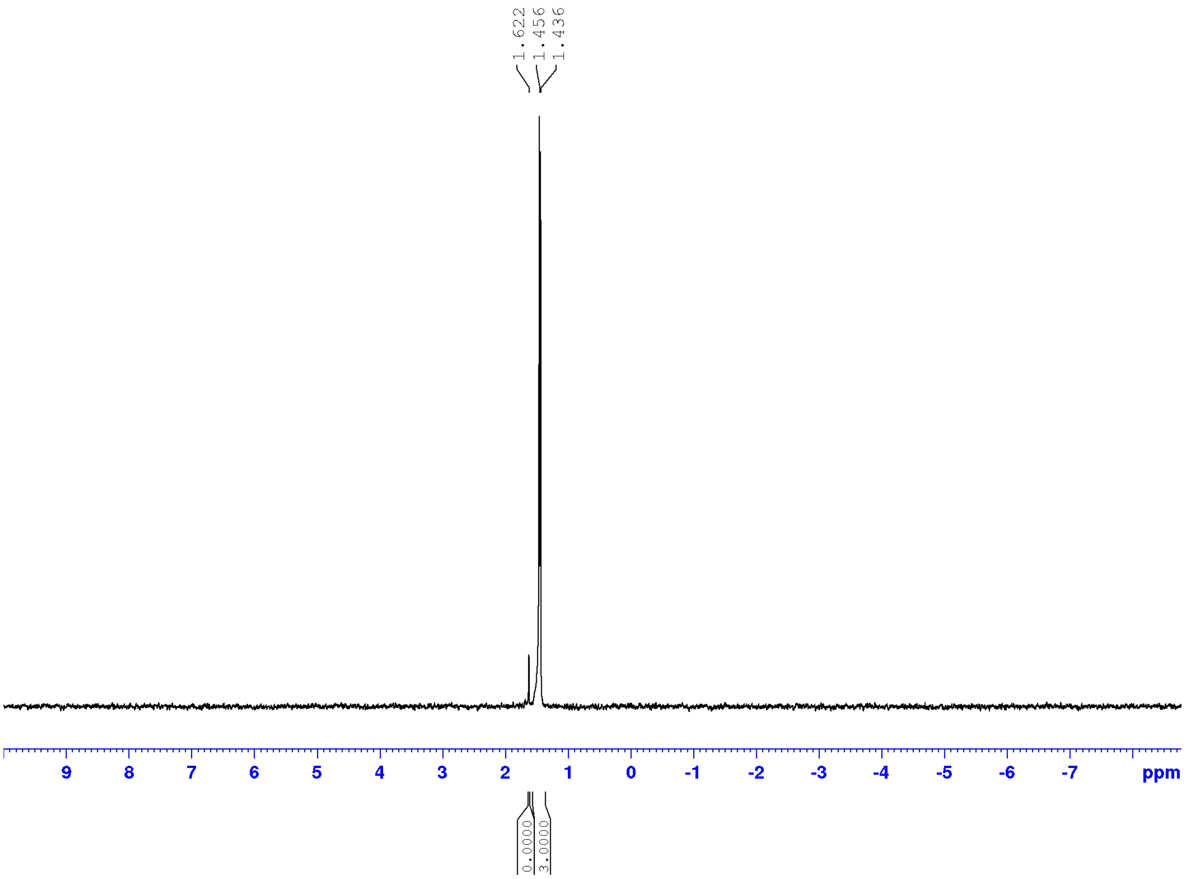

Figure S1. ^{2}H NMR ($CHCl_3$, 76 MHz) spectrum of the label.

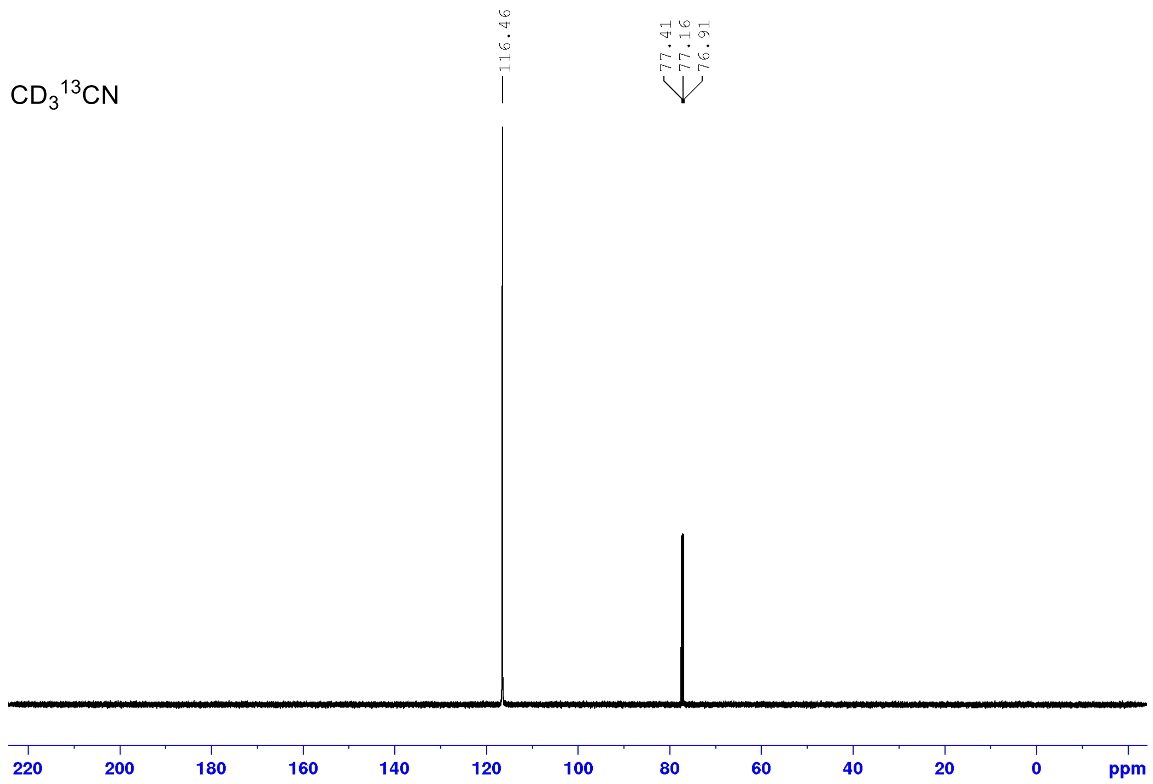

Figure S2. ^{13}C NMR ($CDCl_3$, 100 MHz) spectrum of the label.

S2 ADDITIONAL SPECTROSCOPIC DATA

S2.1 Linear IR Spectra of C≡N Stretch upon Interaction with Salts

KTFSI is the only salt in the current series that has low solubility in acetonitrile (< 0.1 m). K^+ interacts very weakly with the nitrile moiety due to the low charge density of this large monovalent cation. As a result, it does not induce any noticeable perturbation to the solvent C≡N stretch (**Fig. S3**, green). Solubility can be increased severalfold by forcing the cation in solution via coordination with 18-crown-6 macrocycle, which however does not fully enclose the cation, but only coordinates it equatorially leaving polar regions for interaction with the solvent. Nevertheless, even in this case no noticeable changes occur in the C≡N stretch spectral region (**Fig. S3**, blue). In contrast, **Fig. S4** shows the significant influence of the other salts on the nitrile resonance of CD_3CN due to the cation coordination.

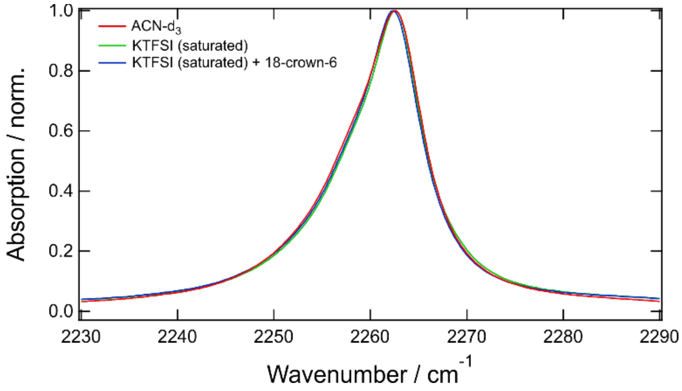

Figure S3. IR spectrum of C≡N stretch of neat CD_3CN (red), saturated solution of KTFSI (< 0.1 m, green), and saturated solution of KTFSI in presence of excess of 18-crown-6 (> 0.25 m, blue).

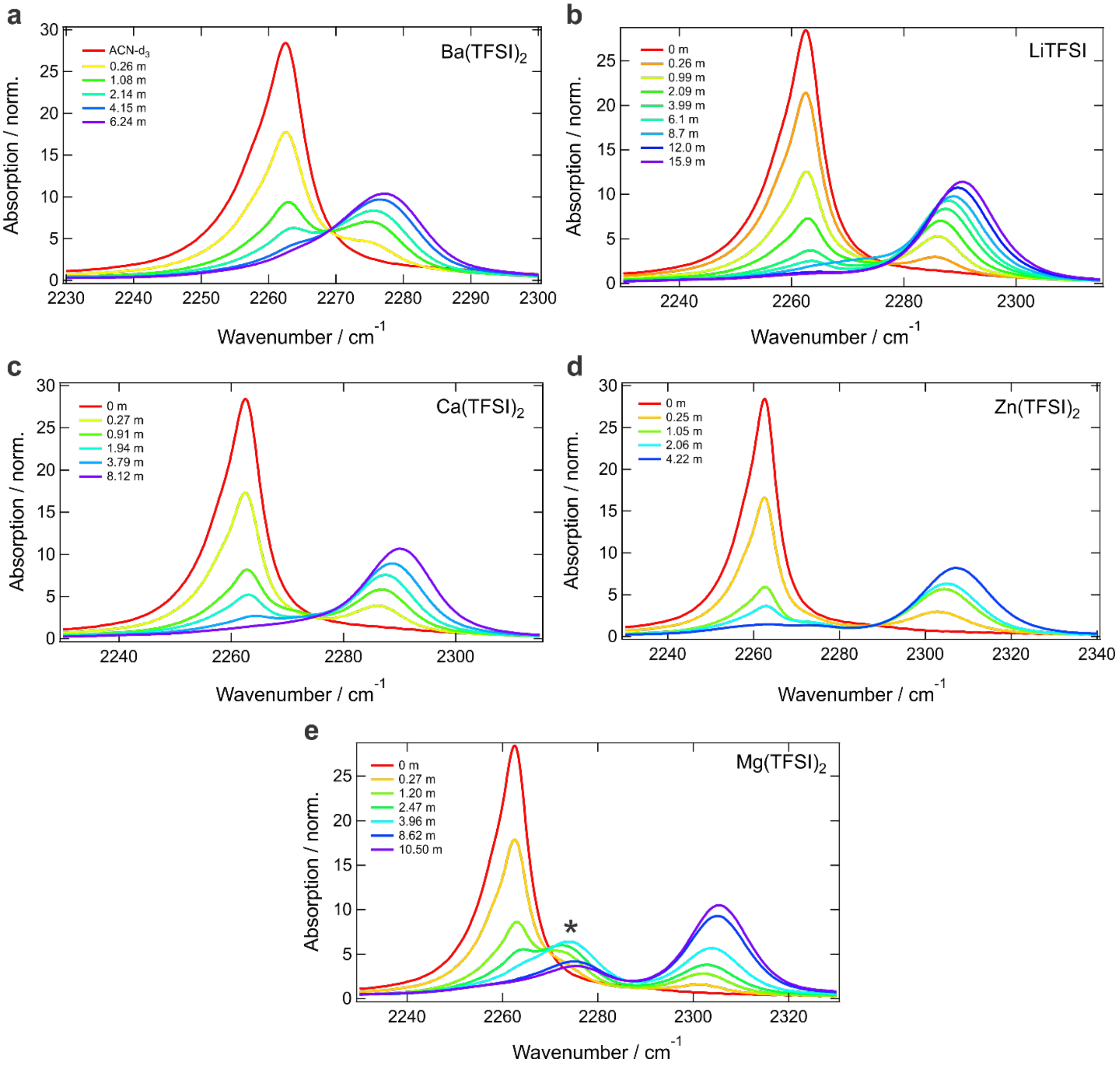

Figure S4. IR spectra in the solvent C≡N stretch region of CD_3CN upon addition of various amounts of $M(TFSI)_n$ salts of increasing cation charge density: Ba^{2+} (**a**), Li^{+} (**b**), Ca^{2+} (**c**), Zn^{2+} (**d**), Mg^{2+} (**e**). Asterisk denotes the nitrile H-bonded to metal-coordinated water (**Section S2.4**). All spectra are normalized to the absorption band of the symmetric C–D stretch mode at 2115 cm^{-1}.

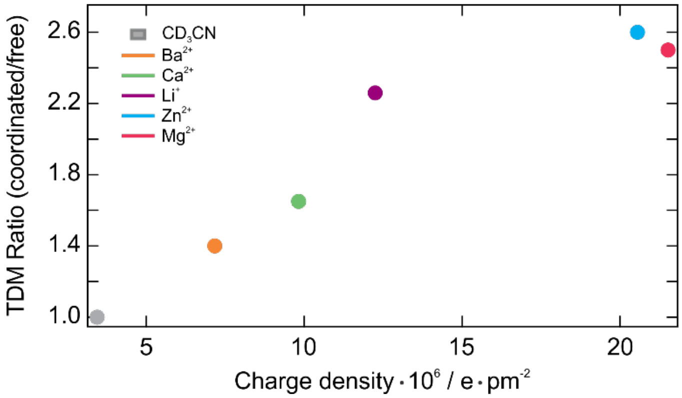

Figure S5. Scaling of the transition dipole moment (TDM) of the metal-coordinated C≡N stretch of CD_3CN with the charge density of the cation.

S2.2 Fermi Resonance in the Label

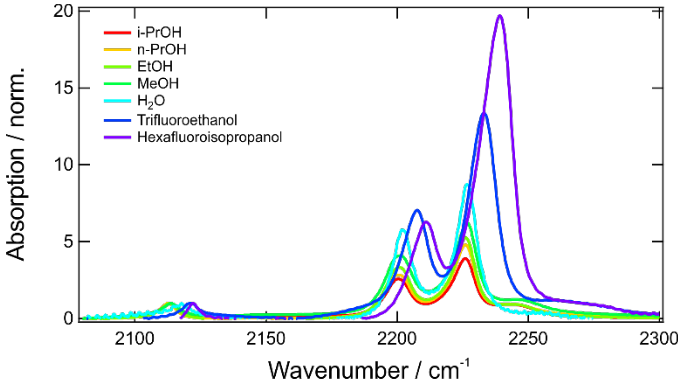

Figure S6. Exemplary IR spectra of the dilute 3% $CD_3{}^{13}CN$ label in the C≡N stretch region in H-bond donating solvents. All spectra are normalized to the C–D symmetric stretch band at 2112-2122 cm^{-1}.

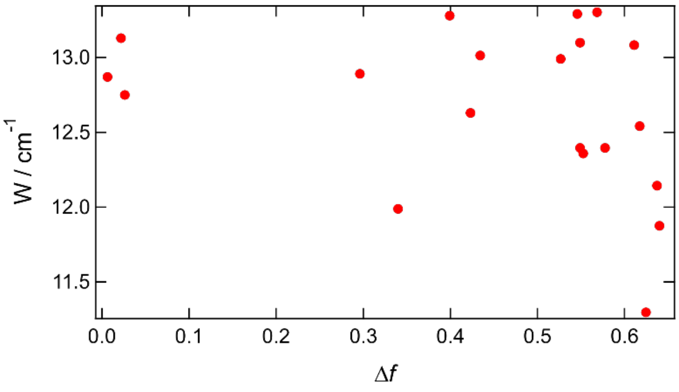

Figure S7. W as a function of Onsager polarity function, $\Delta f = f(\varepsilon) - f(n^2)$, where $f(x) = \frac{2(x-1)}{2x+1}$.

S2.3 Vibrational modes of various acetonitrile isotopologues in the C≡N stretch region

Table S2. Computational and experimental results for the vibrational modes of various acetonitrile isotopologues in the 2100-2300 cm^{-1} spectral region. Computed anharmonic frequencies and intensities are obtained at ωB97XD/6-311++G(d,p) level of theory with PCM solvent model of acetonitrile without capturing possible Fermi resonances. The oscillator strength of the experimentally measured vibrational bands of each isotopologue is referenced to the most intense transition in the region of interest.

**Calculated**			
**Isotopologue**	**Frequency / cm^{-1}**	**Oscillator Strength / km/mol**	**Nature of the mode**
CH_3CN	2263	43.5	C≡N stretch
$CH_3{}^{13}CN$	2198<br>2225	23.5<br>20.1	C≡N stretch<br>C-C stretch+C-H bend
$CH_3C^{15}N$	2205<br>2238	5.6<br>38.1	C-C stretch+C-H bend<br>C≡N stretch
$CH_3{}^{13}C^{15}N$	2173<br>2212	33.4<br>8.6	C≡N stretch<br>C-C stretch+C-H bend
CD_3CN	2263	43.9	C≡N stretch
$CD_3{}^{13}CN$	2212	41.3	C≡N stretch
$CD_3C^{15}N$	2239	41.1	C≡N stretch
$CD_3{}^{13}C^{15}N$	2185	36.3	C≡N stretch
${}^{13}CD_3{}^{13}CN$	2148<br>2151<br>2208	0.17<br>0.10<br>42.0	C-D stretch<br>C-D stretch<br>C≡N stretch
${}^{13}CD_3{}^{13}C^{15}N$	2147<br>2151<br>2181	0.05<br>0.05<br>40.8	C-D stretch<br>C-D stretch<br>C≡N stretch
**Experimental**			
**Isotopologue**	**Frequency / cm^{-1}**	**Relative oscillator strength**	**Nature of the mode**
CH_3CN	2203<br>2253<br>2293	0.02<br>1.00<br>0.22	^{13}C≡N stretch*<br>C≡N stretch<br>C-C stretch+C-H bend
CD_3CN	2116<br>2198<br>2214<br>2263	0.03<br>0.01<br>0.03<br>1.00	symm. C-D stretch<br>C-D bend overtone<br>C-D stretch<br>C≡N stretch
$CD_3{}^{13}CN$	2116<br>2198<br>2224<br>2251	0.07<br>1.00<br>0.81<br>0.14	symm. C-D stretch<br>C-D stretch<br>C≡N stretch<br>unidentified
${}^{13}CH_3{}^{13}CN$	2202<br>2260	1.00<br>0.08	C≡N stretch<br>C-C stretch+C-H bend

*due to the natural abundance of the ^{13}C isotopologue present as an admixture

S2.4 Influence of Water on IR Spectra of Solvent C≡N Stretch

The salts were used as received and they contain small but different amount of water depending on their hygroscopicity. $Mg(TFSI)_2$ is the most hygroscopic salt that contained significant amount of water coordinated to Mg^{2+} cation. It leads to the appearance of the distinct peak around 2275 cm^{-1} between the peaks of the free and Mg^{2+}-coordinated ACN ascribed to the water-mediated Mg^{2+} complex (see main text). **Fig. S8** demonstrates the O–H stretch region of the solutions shown in **Fig. 3b** (main text) using the same color code. Clearly, even in nominally anhydrous 1 m $Mg(TFSI)_2$ in acetonitrile, there is a notable O–H stretch absorption peak of coordinated water that grows upon addition of small amounts of H_2O. **Fig. S9** demonstrates that a prolonged lyophilization of the superconcentrated 10.5 m $Mg(TFSI)_2$ solution removes part of the water content leading to the decrease of the water-mediated Mg^{2+} species with a concomitant increase of the ACN directly bound to Mg^{2+}, and a distinct appearance of the band of the metal-uncoordinated ACN surrounded by TFSI-anions at 2257 cm^{-1} that is otherwise to a large extent hidden by the H-bonded species. Even after several consecutive prolonged lyophilizations, the water-mediated species cannot be removed completely.

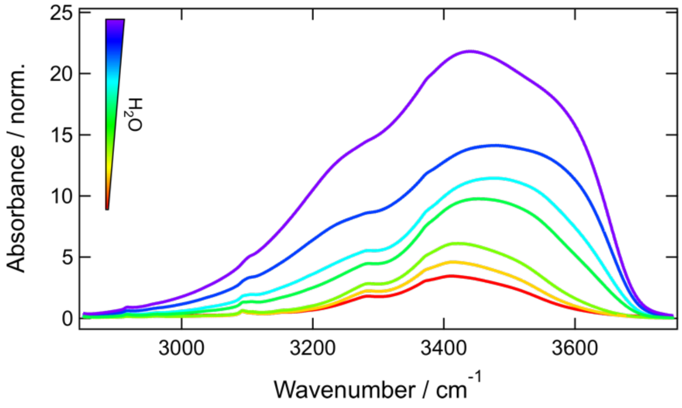

Figure S8. IR spectra in the O–H stretch spectral region upon addition of small amounts (~1-10% v/v) of water to 1 m $Mg(TFSI)_2$ CD_3CN solution (red spectrum). The solutions and color code are the same as in **Fig. 3b** in the main text. The spectra are normalized to the weak C–D symmetric stretch band around 2216 cm^{-1}.

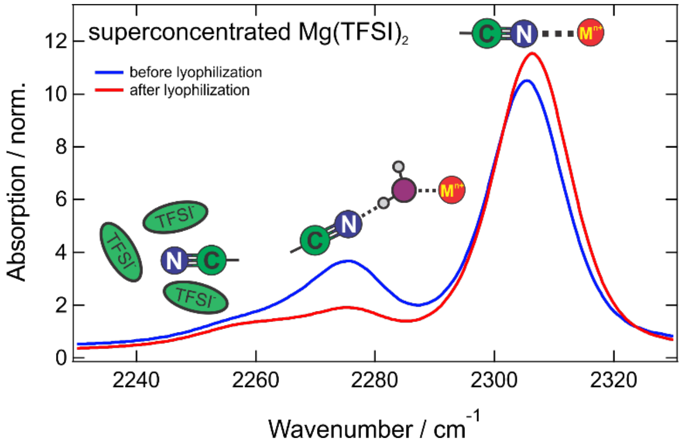

Figure S9. IR spectra in the solvent C≡N stretch region of the superconcentrated 10.5 m $Mg(TFSI)_2$ before (blue) and after (red) the lyophilization. The spectra are normalized to the absorption band of the symmetric C–D stretch mode at 2115 cm^{-1}.

The peculiar influence of water on the IR spectrum of Mg^{2+}-coordinated $CD_3{}^{13}CN$ label has to be discussed in detail. Both Mg^{2+} and Zn^{2+} induce large frequency upshift of the $^{13}C≡N$ stretch and decrease the Fermi coupling constant (see main text) such that the FR is completely suppressed in the Mg^{2+}/Zn^{2+}-coordinated label. Therefore, metal-bound $CD_3{}^{13}CN$ features a single $^{13}C≡N$ stretch peak around 2255 cm^{-1} with no coupled C–D stretch mode. This is confirmed by the early-time 2D IR spectrum that showcases a diagonal bleach signal at 2255 cm^{-1} and no cross-peaks to the lower-frequency C–D bands (**Fig S10a**). At the same time, there is a distinct pattern of peaks between 2190-2237 cm^{-1} that can be ascribed to the free label and water-mediated Mg^{2+}-$CD_3{}^{13}CN$ species. The position of peaks of the free label are well understood: 2198 and 2225 cm^{-1} (**Fig 4a**). The appearance of the two peaks around the same but slightly upshifted frequencies (2205 and 2230 cm^{-1}) under superconcentration, where no free solvent is expected suggests that the water-mediated species features bands at similar spectral positions. The cross-peaks between these two bands in the early-time 2D IR spectrum demonstrate that indeed these two peaks represent two coupled transitions (**Fig S10a**). The lower 2205 cm^{-1} band is a C–D stretch, and the higher 2230 cm^{-1} band is a C≡N stretch of the water-mediated Mg^{2+}-$CD_3{}^{13}CN$ species. Thus, the Fermi resonance is preserved in this species. Moreover, the 2D IR spectrum at the intermediate concentration where both free and water-mediated label molecules are present in comparable quantities allows to unambiguously resolve their C–D stretch bands (**Fig. S10b**). At early waiting time, there are two distinct diagonal peaks at 2198 (C–D stretch of free $CD_3{}^{13}CN$) and 2205 cm^{-1} (C–D stretch of water-mediated Mg^{2+}–$CD_3{}^{13}CN$) with no cross-peaks between them as expected for two separate species (**Fig. S10b)**. This pattern transforms into a distinct square featuring two additional cross-peaks between the two transitions at late waiting time (**Fig. S10c)**. At the same time, at early time, there is an intense cross-peak to the higher-frequency C≡N stretch at 2215-2237 cm^{-1} detection frequencies (**Fig S10b**). Unlike the C–D stretch blob, this feature cannot be resolved into two bands corresponding to the C≡N stretches of the free label and its water-mediated complex, but the broad diagonal elongation suggests that there are two very closely spaced bands in that region. The respective diagonal bleach at 2215-2235 cm^{-1} (**Fig. S10a**) is also broad as it conceals these two bands. Therefore, it is clear that separation between the free and water-mediated $^{13}C≡N$ stretch is quite small and is only several cm^{-1} in magnitude.

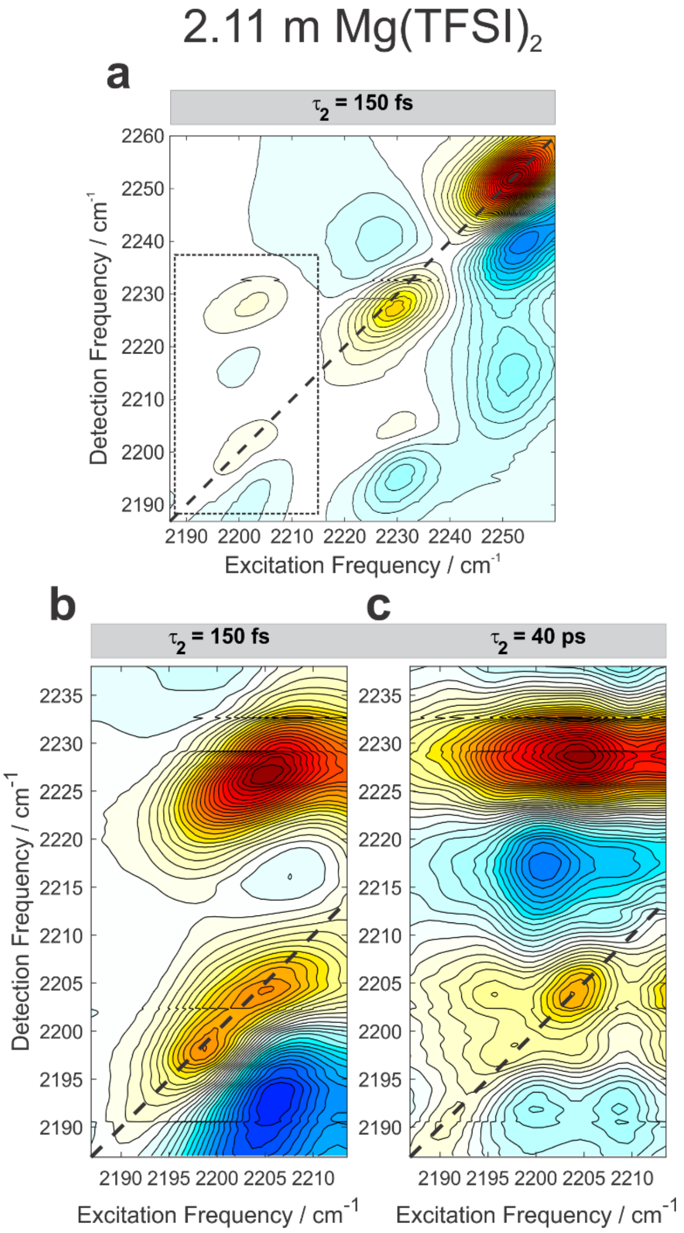

Figure S10. 2D IR spectroscopy of 2.11 m $Mg(TFSI)_2$ $CD_3{}^{13}CN$ solution. **a.** Early waiting time full 2D IR spectrum of this electrolyte solution. **b-c.** Magnified C–D stretch portion of the spectrum shown as a dashed rectangle in panel **a** at early (**b**) and late (**c**) waiting times.

With this knowledge, we can decompose the linear IR spectrum of the label in presence of Mg^{2+} ions and water to understand its non-obvious structure (**Fig. S11**). The spectrum in **Fig. S11** is fitted to disentangle the contributions of all three species present in the solution (free label, water-mediated complex and Mg^{2+}-coordinated label). It is important to apply the maximum number of constraints to reduce the ambiguity of the fit. The lineshape parameters for the two bands of the free label (peak positions, widths and intensity ratio) were fixed to the values obtained from the fitting of the IR spectrum of the neat label without any salt. The parameters of the metal-complexed label around 2256 cm^{-1} can be fixed as well or left floated. They do not affect the quality of the fit or the values for the metal-mediated water complex because this

band is well separated from the rest. The peak position of the C–D stretch of the water-mediated nitrile is fixed to the value obtained from the 2D spectrum (**Fig. S10b**). As a result, only the width and intensity of this band are floated. For the C≡N stretch of the water-mediated complex, all three parameters are allowed to vary.

The two bands obtained from the fit for the water-mediated complex (**Fig. S11**) are subjected to the following consistency check. The H-bond donating ability of the water coordinated to Mg^{2+} is significantly higher than that of free water. According to the dependence of the Fermi coupling constant on the strength of H-bonding to the nitrile group (**Fig. 5d** in the main text), it is expected that the Fermi resonance should be weakened in the water-mediated complex, and the value of W should be even lower than that in neat H_2O (W = 11.9 cm^{-1}). With the ratio of the integrated band intensities and their spectral splitting, by using Eqs. (8) and (9) from the main text we obtain W = 10.3 cm^{-1} in the water-mediated complex. This value is noticeably lower than for $CD_3{}^{13}CN$ in water with no salt present (i.e., non-coordinated water). Moreover, from the general dependence presented in **Fig. 5d** we can estimate that such a coupling constant should correspond to a very strongly H-bond donating O–H moiety. This aligns with the notion that Mg^{2+} coordination strongly activates an O–H fragment of the water molecule and drastically increases its H-bond donating ability.

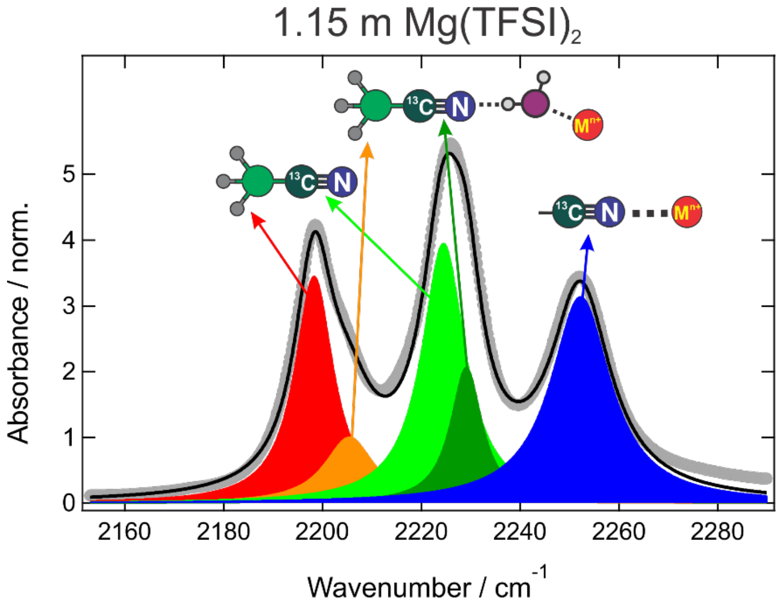

Figure S11. Exemplary decomposition of the linear IR spectrum of 1.15 m $Mg(TFSI)_2$ in $CD_3{}^{13}CN$. Band assignments are indicated with arrows. The spectrum is normalized to the absorption band of the symmetric C–D stretch mode at 2115 cm^{-1}.

It is interesting that H-bonding to the coordinated water should blueshift the C≡N stretch frequency significantly like in CD_3CN (**Fig. 3a-b** in the main text), but strong H-bonding perturbs the FR such that the splitting between the C≡N and C–D stretches decreases, and it causes the redshift of the C≡N band. As a result of these two opposing effects, the C≡N stretch in the water-mediated label complex is upshifted only by 5 cm^{-1} compared to 12 cm^{-1} in the analogous complex of CD_3CN. As a result, the two C≡N bands of the free and water-mediated $CD_3{}^{13}CN$ are ill-separated and visually appear as a single broad band centered at 2226 cm^{-1} in the linear spectrum. All the data are thus described self-consistently.

S3 SUPPLEMENTARY REFERENCES

(S1) Nguyen, L.; Walters, A.; Margulès, L.; Motiyenko, R. A.; Guillemin, J.-C.; Kahane, C.; Ceccarelli, C. Extension of the Millimeter- and Submillimeter-Wave Spectral Databases of Deuterated Methyl Cyanides (CH_2DCN and CHD_2CN). *Astron. Astrophys.* **2013**, *553*, A84. https://doi.org/10.1051/0004-6361/201220088.

(S2) González-Antuña, A.; Rodríguez-González, P.; García Alonso, J. I. Determination of the Enrichment of Isotopically Labelled Molecules by Mass Spectrometry. *J. Mass Spectrom.* **2014**, *49*, 681–691. https://doi.org/10.1002/jms.3397.
